# Mass Spectrometry Imaging of Biomaterials

**DOI:** 10.3390/ma16186343

**Published:** 2023-09-21

**Authors:** Paulina Kret, Anna Bodzon-Kulakowska, Anna Drabik, Joanna Ner-Kluza, Piotr Suder, Marek Smoluch

**Affiliations:** Department of Analytical Chemistry and Biochemistry, Faculty of Materials Science and Ceramics, AGH University of Krakow, A. Mickiewicza 30, 30-059 Krakow, Poland; pkret@agh.edu.pl (P.K.); anna.bodzon-kulakowska@agh.edu.pl (A.B.-K.); drabik@agh.edu.pl (A.D.); nerkluza@agh.edu.pl (J.N.-K.); piotr.suder@agh.edu.pl (P.S.)

**Keywords:** mass spectrometry imaging, MSI, SIMS, MALDI, DESI, biomaterials

## Abstract

The science related to biomaterials and tissue engineering accounts for a growing part of our knowledge. Surface modifications of biomaterials, their performance in vitro, and the interaction between them and surrounding tissues are gaining more and more attention. It is because we are interested in finding sophisticated materials that help us to treat or mitigate different disorders. Therefore, efficient methods for surface analysis are needed. Several methods are routinely applied to characterize the physical and chemical properties of the biomaterial surface. Mass Spectrometry Imaging (MSI) techniques are able to measure the information about molecular composition simultaneously from biomaterial and adjacent tissue. That is why it can answer the questions connected with biomaterial characteristics and their biological influence. Moreover, this kind of analysis does not demand any antibodies or dyes that may influence the studied items. It means that we can correlate surface chemistry with a biological response without any modification that could distort the image. In our review, we presented examples of biomaterials analyzed by MSI techniques to indicate the utility of SIMS, MALDI, and DESI—three major ones in the field of biomaterials applications. Examples include biomaterials used to treat vascular system diseases, bone implants with the effects of implanted material on adjacent tissues, nanofibers and membranes monitored by mass spectrometry-related techniques, analyses of drug-eluting long-acting parenteral (LAPs) implants and microspheres where MSI serves as a quality control system.

## 1. Introduction

MSI’s emerging importance is related to its powerful analytical abilities to measure the distribution of molecules on the surface of a variety of materials. That brings a very broad view of the nature of the processes happening on the surface of the material of interest. The importance of these processes is particularly crucial in the case of in vivo performance of biomaterials. Surface properties of biomaterials obviously can be analyzed not only by MSI methods, but many others can also serve in this field. A typical example could be given: atomic force microscopy (AFM) [1,2], Raman spectroscopy [3,4], X-ray photoelectron spectroscopy (XPS) [5,6], energy dispersive X-ray spectroscopy (EDX) [7,8], Fourier transform infrared spectroscopy (FT-IR) [9,10] or scanning electron microscopy (SEM) [11,12,13]. In fact, several of the above-mentioned methods are simultaneously used to obtain reliable results. MSI can visualize detected molecules with high molecular specificity. Additionally, mass spectrometry techniques can provide structural information about analyzed molecules by application of routinely used MS/MS mode. All MSI methods are continuously improved in terms of sensitivity and spatial resolution, as well as sample application, which is extremely important in the case of imaging experiments. At this point, it is worth emphasizing that obtaining high-resolution images requires an interface capable of analyzing the sample with high spatial resolution. The higher the spatial resolution is, the less material ends up in the mass spectrometer. For this reason, the sensitivity of the mass spectrometer must be as high as possible, which will ultimately allow for increased spatial resolution of the analysis and finally receive a more informative image. In the next Chapter (2) MSI methods applied for the imaging of biomaterials will be described. In addition to the methods described here, there are also other imaging techniques in mass spectrometry, but they have not gained popularity in the field of biomaterials analysis (i.e., LAESI, LA-ICP-MS) [14,15].

The aging of the population and its associated consequences, and the search for better and more effective treatment methods, mean that scientists increasingly turn to biomaterials. These carefully designed objects may bring additional value to the treatment. They may serve not only as spare parts needed such as artificial hip joints, but also as a provider of substances and drugs that may facilitate the healing process. Such complex items need careful examination and testing before being considered a medical product. Here, not only the control of the production process is necessary, but also the careful examination of the influence of such material on the surrounding tissue is crucial. Mass spectrometry imaging techniques, with their ability to indicate the measured sample’s chemical composition, are extraordinary tools for this purpose. They are increasingly used in this context as well as in quality control when new kinds of materials, such as modified membranes or nanofibers are produced.

Since the subject of biomaterials is very crucial and interesting in our review, we decided to trace the reports on the use of MSI techniques to analyze these complex materials. The last review on this subject was conducted in 2017 [16] by Paine et al., and it was slightly more, as the authors admit, materials-chemistry-focused, especially in the case of MALDI applications. Our review also gathers the latest studies in the field and is much more medically-oriented. Articles found in the Internet resources describing various aspects of biomaterials research were sorted into several parts according to the addressed topic.

The first part (Section 3) is devoted to biomaterials used to treat diseases related to the vascular system. New models used in this field, the analysis of lipids and proteins deposition on the grafts, and the analysis of stents that are designed to elute active substances that facilitate the healing process were discussed. In this last case, the ability to visualize the distribution of active drugs by MSI is crucial. The second part (Section 4) goes deeply into the problems connected with bone implant biomaterials. Here, the application of MSI allows simultaneous visualization of mineralized and non-mineralized bone tissue, as well as implanted biomaterials, bone-implant interphases, and eluted active substances including bioactive metal ions. In this field, ToF-SIMS is a technique of choice especially when we consider metal ions. MSI allows us to analyze how implanted biomaterials influence adjacent tissues and such studies were gathered in the next part (Section 5). Biomaterials used to facilitate the cell culture in vitro are somewhere between implants and designed materials such as nanofibers and membranes, which is why they are mentioned in part (Section 6). Here, MSI serves more as an analytical technique that allows for quality control for prepared surfaces that facilitate cell growth. The next part (Section 7) discusses the role of MSI in the analysis of drug-eluting long acting parenteral (LAPs) implants and microspheres. The last two parts (Section 8 and Section 9) are devoted to modified nanofibers and membranes, where MSI serves as a system for quality control that enables checking the correctness of chemical modifications applied by inject printing and plasma lithography and the examination of membrane chemical structure and the absorption of blood-related biomolecules on the modified surfaces.

## 2. Techniques Used for Biomaterial Analysis

### 2.1. Secondary Ion Mass Spectrometry (SIMS)

**Figure 1:**
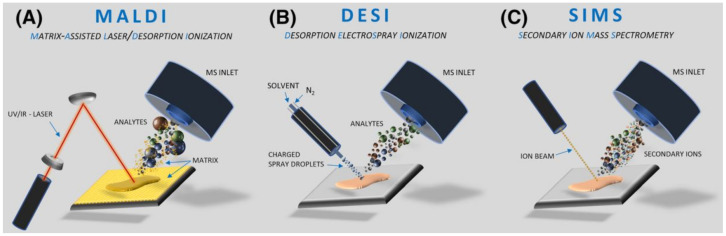
Schemes of major ionization sources applied for biomaterial analyses. Reprinted with permission from John Wiley and Sons [23]. (**A**) MALDI; (**B**) DESI; (**C**) SIMS.

SIMS was the first MS-based technique applied for imaging and still is the most popular in that field, particularly in ToF-SIMS mode (Time-of-Flight Secondary Ion Mass Spectrometry). Several reviews have been written describing the instrumentation, applications, and capabilities of this technique [16,17,18]. The principle of SIMS is analyte bombardment with short series of primary ions (like Ga^+^, Cs^+^, Au^+^, and In^+^), ion clusters or electrons, leading to the ejection from the surface of the secondary ions, molecular fragments, and electrons [19]. Typically, protonated, or deprotonated ions, adducts, or radicals are detected based on their *m*/*z* (mass-to-charge) ratio. SIMS requires placing the flat sample in a high vacuum chamber. The sample is mounted on conductive slides, such as silicon, steel, or indium-tin oxide coated glass (ITO) to avoid surface charging which can have a negative impact on spatial and spectrum resolution, and the overall sensitivity of the analysis. The newest generation of SIMS instruments can achieve a mass resolution of up to 50,000 and a spatial resolution of 50 nm [20]. The high spatial resolution of SIMS is related to source construction enabling extremely high focusing of ions heating the analyzed surface. No other MSI-based imaging techniques can offer such good parameters and that is the main advantage of SIMS over other MSI approaches for the analysis of biomaterials. SIMS is the optimal method for elemental and inorganic analyses, as it can desorb covalently or ionically bound material and also penetrate analyte material in the depth of a few layers. In the case of SIMS quite problematic can be high in-source fragmentation, but high demands to detect larger molecules with this technique put a lot of pressure to improve this source in this term. As a consequence, some improvements in SIMS sources are observed [20]. The advantages and drawbacks of SIMS compared to two other main mass spectrometry imaging techniques, MALDI and DESI are collected in Table 1, and the source schematics for those ionization techniques are shown in Figure 1.

**Table 1:** Comparison of the main features of ToF-SIMS, MALDI, and DESI in imaging mass spectrometry.

	SIMS	MALDI	DESI
Maximum spatial resolution	0.05 μm [20]	5 μm [21]	10 μm [22]
Sample preparation required	minimal	matrix	minimal
Source fragmentation	yes	no	no
Sensitivity	the highest	high	medium
Analysis conditions	vacuum	vacuum	ambient

### 2.2. Matrix-Assisted Laser Desorption/Ionization (MALDI)

MALDI requires the application of a matrix on the surface of the analyte to allow for optimal desorption/ionization process and smooth ions transfer to a mass spectrometer, although matrix-free direct desorption sources exist like DIOS, NIMS, or NAPA [24,25,26]. Sources which do not require matrix application are less popular due to their lower versatility compared to standard sources. MALDI is usually used for large molecules (i.e., proteins), but it works also well for smaller organic compounds and sporadically can be employed for inorganic materials [27]. The principle of MALDI is mixing the sample with the matrix, co-crystallization at ambient conditions, and loading the sample into a vacuum, where the molecules are desorbed and ionized with the laser beam (see Figure 1A). A modified version of this technique, AP MALDI (Atmospheric Pressure Matrix-assisted laser desorption/ionization) is optional, but markedly less commonly used, mainly due to its lower sensitivity [28]. MALDI imaging usually requires flat and thin samples to ensure equal desorption/ionization conditions within the entire analyzed surface. ITO-coated glass slides for sample deposition are recommended to reduce surface charging. High-quality MALDI images require precise matrix application, recognized as the crucial step for obtaining reliable results. Compared to SIMS, MALDI is a less sensitive method but, as it is a soft ionization technique, mainly protonated molecules with minimal fragmentation are observed, which leads to the generation of easier interpretable mass spectra.

### 2.3. Desorption Electrospray Ionization (DESI)

SIMS and MALDI play a major role in MSI imaging, but some biomaterials analyses were also performed by alternative approaches. The most useful seems to be desorption electrospray ionization developed in the early 21st century [29]. DESI can be a complementary technique to SIMS and MALDI mainly due to one reason the sample for analysis is deposited at ambient conditions, so it has the potential to analyze non-vacuum compatible samples (i.e., containing water). Theoretically, DESI is shown as a simple and easy-to-use technique, but in fact, for more complex samples it requires a lot of time for source optimization [30]. In general, DESI is related to well-known electrospray (ESI), but in this case, the sample is deposited at a flat surface and hit by a continuous stream of charged droplets of solvent. As a result, a sample is extracted and desorbed (DESI, see Figure 1B). The typical spatial resolution for DESI in the range of 100 μm is markedly lower than SIMS or MALDI, although even a 10 μm resolution was also reported [22].

## 3. Mass Spectrometry Imaging of Biomaterials in the Vascular System: Models and Therapies

Atherosclerosis and coronary artery disease, along with other heart and vascular system-related diseases, are one of the major causes of serious health problems in the human population worldwide. If untreated, they usually lead to serious complications and death in the majority of patients. Even coronary artery disease, considered alone, is responsible for almost half of sudden cardiac deaths in the population under 50 [31]. The majority of damaged arteries could be replaced using the achievements of modern surgery combined with implants invented by materials sciences. However, to design and test the materials to be implanted into the human organism, to optimize the surgical procedures, or to provide reasonable analytical strategies, and to supervise the interaction between the organism and the implanted material, MSI techniques were successfully introduced.

Currently, the investigations linking MSI and implanted materials used in the damaged vascular system can be divided into three major issues:

(1) Improvement of the models used in the investigations of the vascular and heart diseases;
(2) interactions of the implanted materials with living tissues monitored during the disease progress;
(3) the study of the drug release form drug-eluting stents (DES)

Here, we briefly discuss the scientific improvement provided by the MSI in these categories.

### 3.1. Model Improvement for the Vascular System Diseases

The implantation of the typical bare metal stents (BMS) or bioresorbable vascular scaffolds (BVS) is surgically and therapeutically well recognized [32]. The third type: drug-eluting stents (DES) seems to be the most promising in some circumstances [33]. However, the appropriate and highly reliable models of such a stent’s interaction with tissues are necessary, as there is still a wide room for improvement here. DES links a scaffold and drug release properties. As we currently know, the use of DES is limited to some types of illnesses and there are still important issues with their application, like in the case of diabetic patients [34] or other patient groups, responding by severe calcifications in the implanted area [35]. Based on the reliable investigation models, at least some of the questions of their application could be solved soon. To date, the most prevalent ones use porcine coronary arteries of young animals. Unfortunately, such models have a limited predictive value for DES application, as young animals’ arteries do not mimic atherosclerotic tissue well. Nevertheless, this porcine model is still recognized as a “gold standard” in the evaluation of stenting safety [36]. Even involving animals with atherosclerotic plaque, determined as better for DES testing [37], is associated with some problems related to repeatability, animal availability, overall costs of the experiments, and other issues. Trying to resolve some of these problems, Razzi and co-workers designed an excellent, ex vivo model of atherosclerotic plaque for drug delivery studies [38]. They simulated atherosclerotic plaque in a model of an extracted coronary artery, mounting the lipid-free and lipid-filled hydrogels between the internal artery wall and implanted stent. To obtain reasonable results such a model needs extensive supervision with the aid of analytical techniques. Here, MALDI-MSI was selected as a convenient and fast screening method to check the transport efficiency of everolimus, a drug routinely used in DES implanted into patients undergoing percutaneous coronary interventions [39]. The authors have proven that their approach is advantageous over the techniques used up to date, especially in combination with mass spectrometry imaging.

**Figure 2:**
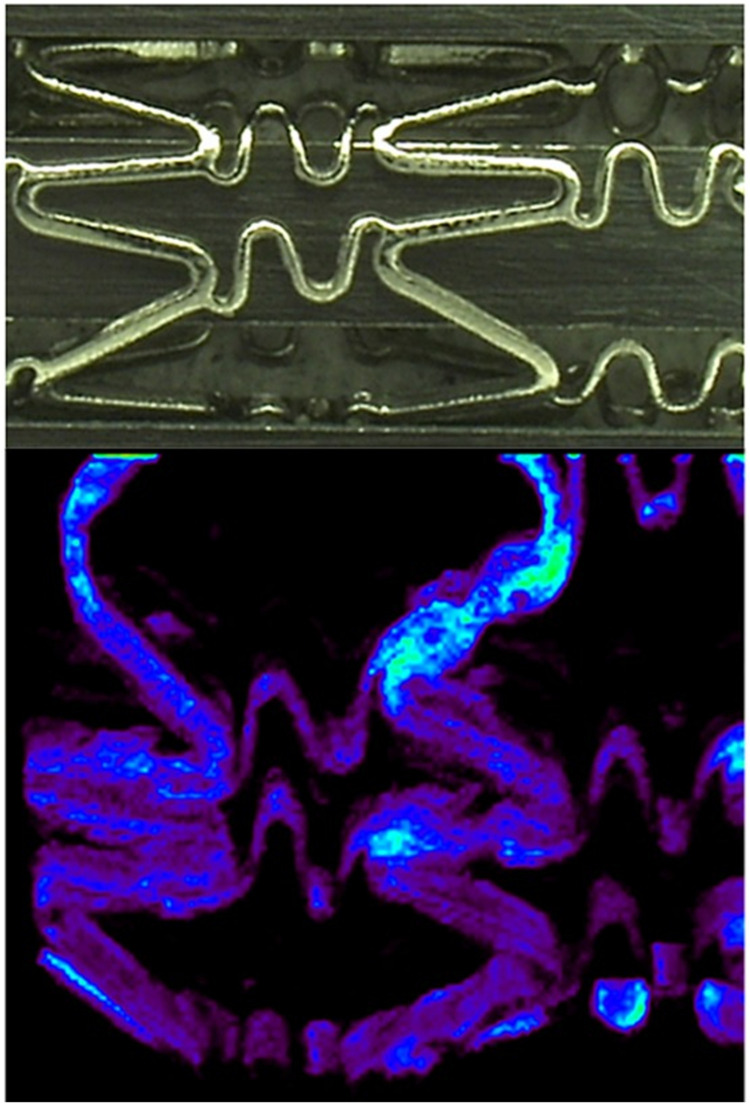
Upper panel: optical image of Cypher^®^ drug eluting stent. Lower panel: MSI of [sirolimus + Na]^+^ on the stent (reprinted from [40] with permission from John Wiley and Sons).

To successfully finalize an exemplary approach shortly described here, extensive optimization of the MSI methods is necessary. This part of the analytical methodologies is usually underestimated by the scientific community; however, technical articles showing the optimization towards special tissues or models should be appreciated, as the presented results spare hundreds of hours of work for analytical systems worldwide. Method development is especially important in MSI techniques, as optimization steps are particularly time-consuming and demanding, due to their multidimensionality. Excellent work has been conducted by Huang et al., where commercially available DES: Cypher^®^ and NEVO™, both from Cordis Corporation, were tested [40]. They focused on the visualization of another typical immunosuppressant drug: sirolimus [41] distribution in stent polymers and its release to the surrounding tissue. Interestingly, instead of the typically applied “one factor at a time” optimization method, routinely used in the available literature [42,43] they used factorial analysis, which allows for changing all necessary factors simultaneously to receive the best possible settings of the analytical system. Moreover, they focused on the 3D stent analysis, which is atypical for the MALDI-MSI approach (see Figure 2). In the optimization, two separate problems were considered: the matrix selection/spraying parameters and the mass spectrometer settings during imaging. In the first problem, five factors were considered, which led to the analyses received from the 35 experiments in total. The second problem was solved by the “single-factor” analysis, where the basic parameters, like laser power and frequency, raster dimensions, and distances between following raster rows were optimized. One of the most important outputs from their investigations is that typical 2D scanning by the MALDI-ToF system can be easily adapted for imaging 3D structures like a stent cage, using the minor modification of the sample holder, which was demonstrated in the imaged samples. The problem of the appropriate mounting of the stent for the successful MALDI-MSI analysis was also described and easily solved elsewhere [44]. The work of Huang et al. also clearly shows that ToF-SIMS, which is recognized as a system of choice for stent analysis [45] could be at least supported, and in some cases even replaced by the easier-to-use MALDI-ToF imaging instrument. ToF-SIMS is sensitive to biological matrix effects and its high level of noise could be an issue in investigating low-concentrated drugs released to surrounding tissues. On the other hand, its superior spatial resolution is difficult or even impossible to overcome by MALDI-MSI.

In fact, up to date, the methodology of vascular stent surface analysis was dominated by ToF-SIMS imaging, usually combined with Raman Spectroscopy [46,47] or with X-ray photoelectron spectroscopy (XPS) [48,49]. A nice example of the approach using ToF-SIMS is a 3D analysis of polymeric coatings used in DES is discussed below. In opposition to MALDI-MSI, “3D” here does not mean imaging of three-dimensionally stretched objects, but rather in-depth analysis of polymer coating with an excellent spatial resolution. It was shown that using special conditions and SIMS sources, even 6.5 μm, the entire depth of the polymer film over the stent was susceptible to the analysis, while the typical depth does not exceed the limit of 1.5–2 μm [49,50]. A similar analysis was conducted by Fisher et al.: they showed some inequalities in sirolimus distribution in PGLA covering a metal stent, using a combination of two ion guns: C_60_^+^ and SF_5_^+^. They also used XPS and confocal Raman spectroscopy as supporting techniques, showing time-dependent dynamics of sirolimus release from the DES surface [48]. The following details, considering in-depth analyses of the DES coatings are given in Section 3.3.

### 3.2. Interactions of the Implanted Materials with Living Tissues Monitored during the Disease Progress

There is a limited quantity of research articles considering the direct interactions between the stents or vascular grafts and the tissue in situ, investigated by the MSI, probably due to limitations of the samples, post-mortem materials availability, or other difficulties. However, at least a few articles touching this problem were found. The work of Fröhlich et al. presents the proteins and lipids adsorption on the biodegradable vascular implants studied by MALDI-MSI [51]. Another article, published by our team, shows lipid deposition in the walls of the artificial vascular graft extracted from the human body, investigated by desorption electrospray (DESI-MS) [52].

In the first mentioned article, an in vivo rat model was used. The primary goal of the analyses was to check protein and lipid deposition onto two polymers typically used for graft manufacturing: expanded polytetrafluorethylene (ePTFE) and thermoplastic polyurethane (TPU). In general, it is known that the profile of lipid and protein deposition in grafts could be related to the risks of post-surgery complications and other long-term risk factors for patients. Such investigations were conducted previously with the aid of other, time-consuming methodologies [53,54]. Here, MALDI-MSI, with the aid of other methods, was used for the characterization of the molecule deposition on both sides (internal/external) of the grafts. It turned out, that just after 10 min from the implantation, there was a significant load of the molecules on both graft sides. Substance profiles were similar in both materials investigated. For the lipid analysis, the observation was interesting, staying also in full agreement with our experiments: cholesterol is one of the major lipids, starting to penetrate graft walls immediately after implantation. After 7 days the graft walls were saturated with this lipid, creating local regions of a high abundance. There was also a general remark that other, less hydrophobic lipids show lesser potential for graft penetration with time.

Our own investigations are based on the unique material available [52]. Polyethylene terephtalate InterGard^®^ vascular graft (Gore Medical, Newark, NJ, USA) was removed from the patient’s body two years after implantation. The reason for removal was an infection, recognized as typical for this material [55] and, as a result, occlusion of the vessel. Our imaging method was desorption electrospray [29], a technique useful especially for lipids imaging in biological tissues [56] if the spatial resolution of the image is not the crucial factor. This opportunity to analyze a unique sample has revealed the real substances deposition in the graft after two years of interaction with the human body. We observed numerous lipids deposition in the graft walls with differentiation to ones prevalently deposited on the outer part, like sphingomyelin 24:1, inner part, like phosphoserine 38:4, phosphocholine 34:1 or penetrating the whole graft wall with additional deposition in the lumen. The most abundant one was cholesterol, forming an atherosclerotic plaque which covered ca. 10% of the graft cross-section.

### 3.3. Characterization of the Drug-Polymer Profile and Drug Elution in DES

Implants equipped with a drug delivery system, in the case of vascular system diseases, are designed to avoid restenosis (subsequent narrowing of the vessel) after placement of an arterial stent. The release rate of the pharmaceutical agent can be controlled by the formulation of the drug/polymer coating and the process of dispersion of the drug in the polymer matrix. The ToF-SIMS technique is applied in this field to study these processes.

Sirolimus (rapamycin) is one of the major anti-restenotic drugs, usually encapsulated in Poly (lactic-*co*-glycolic acid) (PLGA). Three-dimensional (3D) characterization of a drug-eluting stent coating composed of 0 to 50% (*w*/*w*) sirolimus in a PLGA matrix was shown by Mahoney et al. They proved that is possible to use dual beam cluster SIMS (SF_5_^+^ ions for sputtering and Bi^3+^ ions for analysis) for 3D study of polymer drug delivery systems at low temperatures. Coatings in the range of 0–50% (*w*/*w*) sirolimus in PLGA were prepared on two substrates: MP35N metal alloy coupons (metal samples) and BMS. In the obtained SIMS cluster depth profiles, temperature has a significant influence. The best profiles were obtained at −100 °C, which allowed us to study the film at the entire depth reaching up to 6.5 μm. All coatings indicated a drug-enriched surface area, a drug-depleted region, and an area of constant bulk composition. The thickness of the drug’s top layer depended on the drug content. Assembling the raw data into 3D images showed that samples with 5% of the sirolimus were lateral scale homogeneous, with higher values: 25% and 50% showed domain formation in the surface, subsurface, and bulk regions [50].

**Figure 3:**
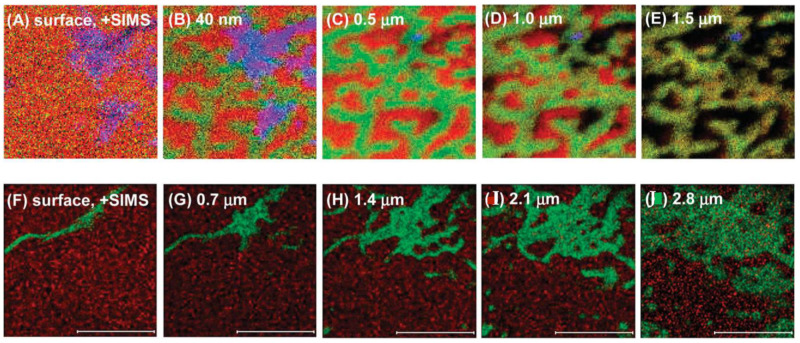
ToF-SIMS 2D images-at-depth were acquired using different sputter ion sources. (**A**–**J**) overlay positive ion mode images. (**A**–**E**) sputtering with SF_5_^+^, sirolimus—red, PLGA nanofiber—green, and K^+^—purple. (**F**–**J**) sputtering with C_60_^+^, images were acquired with 20kV at room temperature, sirolimus—red, PLGA—green. Reprinted with permission from American Chemical Society [48].

Subsequent studies focused on a coronary stent coating containing 25% (*w*/*w*) sirolimus in a PLGA matrix. Information on changes in the 3D distribution of sirolimus in PLGA as a function of elution time was obtained by ToF-SIMS. Drug elution was induced by sample incubation in an eluting buffer and gentle agitation. During the study, an Au^+^ ion beam for analysis and a C_60_^+^ ion beam (higher efficiency than SF_5_^+^) for sputtering were used. Depth profiling monitored the presence of C_5_H_10_N^+^ and CN^−^ secondary ions which are diagnostic for sirolimus, and the presence of the C_3_H_4_O^+^ and C_3_H_5_O_2_^−^ secondary ions which are diagnostic for PLGA. Ion maps of the ToF-SIMS depth imaging are shown in Figure 3. Samples of 25% sirolimus in PLGA were eluted for 1 h and 1 day (time zero for the control sample). Results of testing revealed that a large fraction of the drug (~55%) was eluted on the first day. Both 2D and 3D imaging results show large surface areas, as well as subsurface channels, that consist mainly of the drug, followed by a sirolimus-depleted region, and homogeneous dispersion of the drug in the PLGA matrix. Below the surface, areas composed mainly of PLGA contain a significant amount of sirolimus, but areas composed mainly of the drug contain a very low amount of PLGA. The results of the study showed that elution from the drug-enriched surface occurs in a short time, and more gradually from the subsurface areas. The method of application of the formulation may be of great importance for the early elution rate of the drug from the coating [48].

Another group of researchers examined four formulations containing from 5 to 50 (*w*/*w*) rapamycin (Rap) in PLGA, both with and without a PLGA topcoat (“coating”) at a thickness of 7 μm on coupons and on stents. In this study, applying the ToF-SIMS (SF_5_^+^ sputter source, Bi_3_^+^ acquisition source) confirms the presence of both rapamycin and PLGA through the detection of intact molecular signals and molecular fragments. Formulations containing 5% rapamycin/95% PLGA, 25% rapamycin/75% PLGA, 50% rapamycin/50% PLGA, and 25% rapamycin/75% PLGA with a PLGA cap coat were used. For the sample, 5% Rap/95% PLGA, the signal is maintained in the ToF-SIMS depth profile through the entire thickness of the coating. However, for the samples with higher drug concentrations, there is a problem with maintaining the signal along with the depth. For example, in the 50% Rap/PLGA sample, the drug and polymer signals are observed only up to half the thickness of the sample. Interestingly, samples with a low drug content in the PLGA can be sputtered and analyzed to a greater depth, due to the properties of the matrix. For the films (thin layer of the polymer matrix with the drug), the ToF-SIMS depth profiles show a total depth of up to 500 nm, which means that the drug and PLGA molecular signals stay visible for all four formulations. Moreover, the rapamycin signal shows an enrichment of the drug at the surface for each sample, but the enrichment depth of the rapamycin increases in proportion to the drug concentration. Based on the results the rapamycin enrichment region is about 5 nm for the 5% Rap/95% PLGA, and for subsequent samples is: 35 nm for the 25% Rap/75% PLGA, and 115 nm for the 50% Rap/50% PLGA sample. The near-surface region images for the formulation of the 25% Rap/75% PLGA show the relation between drug/polymer morphology with depth. On the surface, the film consists almost entirely of rapamycin, but when the surface of the sample erodes, heterogeneous drug distribution is observed. For the 25% and 50% Rap samples, the imaging data show the varying size of the polymer/drug domain with depth, which helps to understand the drug release profile of the coatings. In addition, the results suggest rapid drug release at the initial time points [46].

## 4. Mass Spectrometry Imaging of Bone Implant Biomaterials

People worldwide are living longer, and according to WHO, by 2050 the world’s population of people aged 60 years and older will double. The number of humans aged more than 80 years is expected to triple by that time. Aging consequences such as the increase in a wide variety of molecular and cellular damage lead to a decline in physical and mental capabilities, a growing risk of disease, and eventually death. Osteoporosis is an age-related disease, characterized by a reduction in bone density due to loss of bone mineral content, and changes in bone microstructure. These alterations result in instability of bone that can lead to more frequent fractures and consequently to prolonged or incomplete fracture healing. Osteoporotic fractures are a significant health problem worldwide with almost 9 million cases every year, especially at sites like vertebrae, proximal femurs, proximal humeri distal radii, and femur [57].

Bone marrow exists in two different states; the osteogenic form, also termed red bone marrow, and the yellow bone marrow, which develops during bone maturation. Whereas red bone marrow mostly contains hematopoietic cells like blood cells, platelets, mesenchymal stem cells, fibroblasts, adipocytes, osteoblasts, osteoclasts, macrophages, or endothelial cells, the yellow bone marrow is mainly composed of bone marrow adipocytes. The percentage of yellow bone marrow increases up to 70–80% during bone maturation [58]. Bone is a highly vascular, mineralized connective tissue extraordinary for its strength and regenerative capacity. The composition of bone tissue is of great significance for the understanding of fracture healing, bone implant interactions, and mineralization disturbances such as osteoporosis, and Turner’s syndrome. The application of ToF-SIMS in osteoporotic bone research allows simultaneous visualization of mineralized and non-mineralized bone tissue, as well as implanted biomaterials and bone implant interphases [59]. ToF-SIMS enables the simultaneous identification of both organic and inorganic substances within the same sample in a single analysis process in 2D and 3D [58]. With ToF-SIMS the ions distribution in bone cross-sections is mapped semi-quantitatively with a lateral resolution of up to 1 μm [60]. ToF-SIMS was successfully applied for tracking pharmaceuticals in a biomaterial from in vitro to in vivo experiments, as well as for the investigation and imaging of osteoporotic bone in an animal model [61]. Usually, osteoporosis is experimentally induced in animal models by ovariectomy which can be accompanied by a deficiency diet lacking vitamins C, D2, D3, calcium, and phosphorus as well as soy- and phytoestrogen-free [58].

Obtained ion maps are able to reveal the distribution of the organic compounds, as well as the localization of mineralized parts of the bone; however, the quantification of concentrations is challenging, as the ionization process and hence the ion yields strongly depend on the chemical environment of the elements and molecules at the surface. Therefore, appropriate standards with almost identical chemical composition as the analyzed material are required, and the relative fragment mass intensity of the analyte of interest should follow a linear relationship with its concentration in the standard [60]. Nevertheless, some alterations in the ion maps may occur in the case of the bone region adjacent to the implant, indicating that no secondary ions are generated from this area. That can be the reason for the dehydration and shrinking effect of the tissue, thus causing a gap between the implant and tissue. It is also possible that cutting the sample causes some dislocation between the implant and tissue [62].

**Figure 4:**
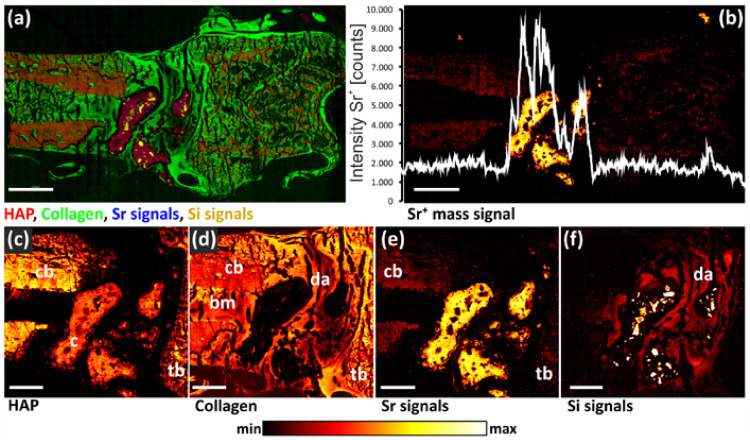
ToF-SIMS mass images of a rat bone section with implanted biomaterials; (**a**) rat bone section with mass fragments of mineralized bone in the form of HAP (hydroxyapatite, red), nonmineralized collagen (green), strontium signals (blue), and silicon signals (yellow); (**b**) distribution of strontium; (**c**) distribution of HAP (mineralized bone) in the remaining bone cement (c), cortical bone (cb), and trabecular bone (tb); (**d**) collagen signals in the former defect area (da), cortical and trabecular bone, as well as bone marrow (bm); (**e**) strontium distribution in the remaining bone cement fragments; (**f**) silicon signals from glass particles in remains of implanted biomaterial. Reprinted with permission from AIP Publishing [61].

In osteoporosis therapy, several drugs are administered systemically to prevent fractures by slowing down the bone resorption process or stimulating bone formation. Numerous drugs, such as antibiotics, osteoanabolic substances, or anti-resorptive drugs can be released into a specific bone defect and locally promote tissue restoration and bone formation, including bioactive metal ions like Sr^2+^, Mg^2+^, Cu^2+^, and Co^2+^. Those metal ions can reduce bone resorption and simultaneously stimulate osteoblastic bone formation, which promotes bone healing and fracture repair [63]. Bone processes are essential for maintaining healthy bones. Studies have shown that systemic osteoporosis therapy with orally administered strontium ions (Sr^2+^) in the form of strontium ranelate has also improved the osseointegration of bone implants (see Figure 4). However, due to the high doses applied, it had to be withdrawn because an increased risk of vascular calcification was observed [64]. Therefore, the main objective of biomaterial studies is to develop new materials modified with substances that are specifically capable of actively contributing to bone fracture healing and osteogenesis, even in systemically altered osteoporotic bone instead of classical, high-dose oral tablet therapy.

To meet the specific requirements of osteoporotic bone fracture healing, when seeking new implant materials, fracture stabilization plays an important role. Material benefits, such as ease in surgical handling, high biocompatibility, resistance to abrasion, small coefficient of friction, and self-lubricating properties are also important [65]. Studies on the drug release and distribution process of therapeutically active Sr^2+^-ions in healthy and osteoporotic bone were performed using ToF-SIMS analysis of metal ions diffusion coefficient [64]. Authors have designed calcium phosphate bone cement enriched with Sr^2+^ to be sustainably released over an extended period in a constant dose and to be distributed in the surrounding tissue with a concentration that is slightly higher than the effect threshold [66]. Different kinetic models for various parameters have been applied to describe the release process fitted with the solution of Fick’s second law for diffusion.

Since Sr^2+^ ions are an effective therapeutic agent for the healing of osteoporotic bone fractures, they are often used in the form of strontium-modified bone cement. Additional studies using ToF-SIMS led to the development of the experimental protocol for transport studies in bovine bone marrow [58]. Additionally, orbitrap secondary ion mass spectrometry (OrbiSIMS) was applied for definite signal identification of lipids and fatty acid species in rat bone marrow [67]. Comprehensive 2D and 3D mass spectrometric imaging analyses, depth profiling, as well as OrbiSIMS spectrometric analysis, discovered faster Sr^2+^ diffusion in rat bone marrow areas correlated with lower intensity of lipid and fatty acid signals than in areas with higher lipid/fatty acid content [58]. Furthermore, the Authors observed that bone marrow fat consists mainly of palmitic acid, stearic acid, oleic acid, triacylglycerides, and phosphatidylcholine. Deviations obtained with the Orbitrap analyzer were for most selected peaks <2 ppm, in comparison to deviation obtained with the ToF analyzer was >45 ppm for inorganic strontium species and >20 ppm for lipid peaks on average. The mass resolution of signals obtained using OrbiSIMS was also higher than the mass resolution obtained with the ToF analyzer [58].

Another research was carried out using strontium surface functionalization of specially designed titanium-niobium (Ti-^40^Nb) implant to accelerate bone growth by stimulating the osteoblasts and, at the same time, to inhibit the osteoclasts, which are responsible for bone resorption [68]. This remodeling process accelerated by the biomaterial may provide an advanced implant system that is mechanically adapted to the altered bone with the ability to stimulate bone formation. Whereas titanium is a well-recognized biomaterial for the production of implants for hard tissue replacement and fracture stabilization, it still needs to be improved to meet the specific challenges that orthopedics and traumatology are facing. The fundamental disadvantages of the currently used titanium implants are their high stiffness mismatch to bone, which could limit implant biocompatibility. It is an important aim to develop advanced metallic implants that are mechanically adapted to the weakened bone structure and ensure fast bone integration by modified surface characteristics. ToF-SIMS analysis of the Ti-^40^Nb surface after the release experiment revealed traces of residual strontium [68].

Furthermore, altering calcium phosphate bone cements might be important for their application in the treatment of large bone defects as presented in the study of trivalent chromium ions incorporated into a biomaterial [63]. ToF-SIMS was applied to investigate the release of Cr^3+^ ions from the cement after implantation and distribution in the implant region and the surrounding bone tissue. Calcium phosphate cement (CPCs) are also very significant implant materials for bone defects because of their osteoconductive, resorbable, and biocompatible characteristics [57]. Furthermore, in osteoporosis, additional stimulating factors like bone morphogenic protein (BMP), vascular endothelial growth factor (VEGF), or transforming growth factor beta (TGF-ß) are necessary for the differentiation of cells into osteoblasts and factors are beneficial for bone regeneration [57]. Additionally, the effect of magnesium oxide that induces bone formation in experimental animal models and stimulates bone formation in vivo was studied by ToF-SIMS [69]. The ToF-SIMS analysis showed molecular information measured in regions from the bone marrow into the cortical bone, that presented the increased thickness of the compact bone induced by MgO implantation. The application of MgO resorbable, biocompatible biomaterial causes stimulation of bone formation and accelerates the remodeling process that can be successfully used for fracture healing [69]. Ultrahigh molecular weight polyethylene (PE-UHMW) has been studied as a material in restoring the acetabular cup in hip joints and cartilage in knee joints [65]. PE-UHMW’s major weakness is shelf-life aging by oxidation, and even surface coating with vitamin E and polyethylene glycol (PEG) consequently leads to in vivo aging. MALDI-MSI was used to characterize polymer-based hip joint explants and PE-UHMW modifications at the molecular level [66]. Sample preparation for MSI analysis of hydrophobic polymer surfaces was developed and various lipid classes crucial for the lubrication process were identified by MS/MS analysis. Ceramic implants were among the initially utilized biomaterials in orthopedic surgery, with zirconia implants frequently used as oral implants. Zirconia has some esthetic benefits compared with titanium particularly in cases of bone resorption, as the white color might be less disturbing if the implant surface is exposed. ToF-SIMS analysis was developed to study differences between ceramic and metallic implant surfaces, and mineralization of bone tissue in the healing process of selected ceramic implant types [70]. The recent development of MSI, especially including ToF-SIMS, enabled monitoring the release and incorporation of active substances into bone, or chemical characterization of biomaterial/bone interfaces, bone cells as well as bone mineral status. Imaging ToF-SIMS has been widely used in the study of bone formation, biomaterial–protein interaction, and implant tissue interactions, mainly due to its high sensitivity to all elements and its high-resolution global imaging capabilities. ToF-SIMS has been optimized to study tissue implanted with different biomaterials with results shown as elemental distributions of Ca^+^, Mg^+^, K^+^, Na^+^, P^−^, CN^−^, PO_2_^−^ and PO_3_^−^ [71].

In addition to the requirements of the biomaterials, the property and characteristics of drug release and distribution/propagation in the tissue play a significant role in the development of new implant materials with optimized functionality used in the treatment of diseases such as osteoporosis might become a growing public health problem with serious socioeconomic consequences.

## 5. Evaluation of Biomaterial Influence on the Adjacent Tissue by Mass Spectrometry Imaging Techniques

The MSI techniques are able to investigate the interaction between the biomaterial and the tissue since they can elucidate the distribution of different molecules inside and around the artificial material. It means that they are able to provide information about how the used material behaves in vivo and what is the reaction of surrounding tissues and the host immune system.

**Figure 5:**
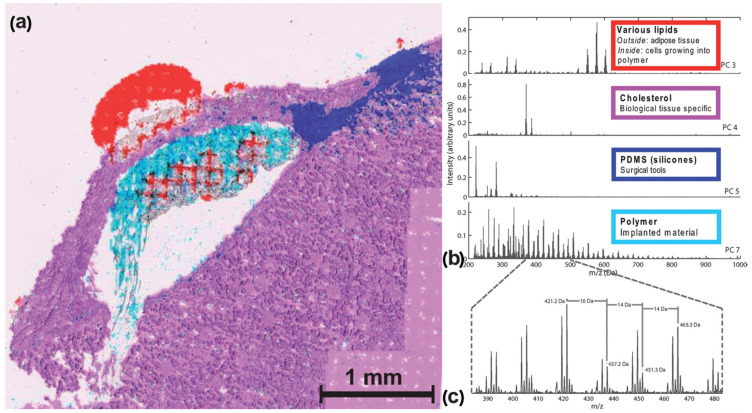
(**a**) Hydrogel implant under the renal capsule of a rat. (**b**) PCA analysis indicated at PC 3—lipids, adipose tissue outside the renal capsule—red, PC 4—cholesterol, a highly abundant cell membrane component—purple, PC 5—silicone contamination—navy blue, and PC 7—the polymer with characteristic PEG monomer unit—blue. (**c**) Shows the PEG distribution in detail with characteristic 16 Da and 14 intervals. Adapted with permission from American Chemical Society [72].

Klerk et al. conducted one of the first studies in this field [72]. In this work, they studied hydrogel drug delivery carriers composed of elongated polymer chains cross-linked by the quadruple hydrogen bonding ureidopyrimidinone group [73]. The biomaterial was implanted under the renal capsule of rats, and the kidneys were collected 15 days after implantation. The kidney tissue slices were covered with gold and measured in the positive and negative secondary ionization modes. PCA analysis was performed to indicate polymer-specific signals and biologically relevant peaks (see Figure 5). Cellular infiltration was indicated into the polymer, and it was direct evidence of the presence of active cells inside the hydrogel. To distinguish between different types of macrophages that may play a vital role in this process, the macrophages were in vitro polarized by six different polarization agents for 6 days. After culturing, the cells were washed with sucrose (300 mM) to remove salts, cytospined on indium tin oxide (ITO) coated glass slides, covered with gold, and measured. The gold-covered and non-covered samples were compared to eliminate the Au adducts.

The results from the cell cultures made it possible to find a few specific peaks in the differently polarized macrophage standards. Unfortunately, a reliable classification was not possible. Nevertheless, the potential of ToF-SIMS imaging in the analysis of biomaterials, and the possibility of using chemical signals to distinguish between cellular and polymer components was proven highly useful.

Hydrogels belong to the class of materials that could be either synthetic (made from molecules such as poly(ethylene glycol)) or based on native proteins, such as collagen or fibrin, and may be used as 3D scaffolds for tissue engineering [74]. It was shown that injectable collagen hydrogels might help restore the myocardium’s mechanical properties and reduce scar size after acute myocardial infarction [75]. Moreover, peptides from collagen may also provide therapeutic advantages [76,77].

In the study of Clift et al. [78] they used an extracellular matrix (ECM) targeted MALDI MSI strategy to investigate the role of collagen-based biomaterials and ECM remodeling in acute myocardial infarction. The main aim was to prevent the formation of non-contractile scar tissue in post-infarcted mice myocardium. The analytical problem was to check which peptide out of the peptide mixture given in the form of a hydrogel in the heart muscles is the most effective in inhibition of undesirable tissue formation. Seven days after myocardial infarction, mice were treated with either a collagen type I (rhCI) or a collagen type III (rhCIII) hydrogel [75]. Two days after treatment, the tissues were harvested. Obtained tissue sections were deglycosylated with PNGaseF, and Collagenase Type III was applied for protein digestion by TM Sprayer M3. Proteins from the ECM were digested and the tissue slices were covered by CHCA. Finally slides for peptide imaging were rapidly dipped (<1 s) in cold 5 mM ammonium phosphate and immediately dried in a desiccator. MALDI-FT-ICR was used for the analysis. The main challenge in this study was to find peptides with characteristic human collagen sequences, among others of mouse origin, to check if peptides from the hydrogel contribute to ECM remodeling and are responsible for improving cardiac function, which using conventional methodologies, is extremely difficult. As the human COL1A1, COL1A2, and COL3A1 collagens were used for injections in the form of the hydrogels, both methods applied (MALDI-MSI and nanoLC-MS/MS) were able to distinguish between peptides found in mice muscle heart from human collagen and their mice homologs without additional labeling (like in case of fluorescent or MRI techniques [79,80]).

**Figure 6:**
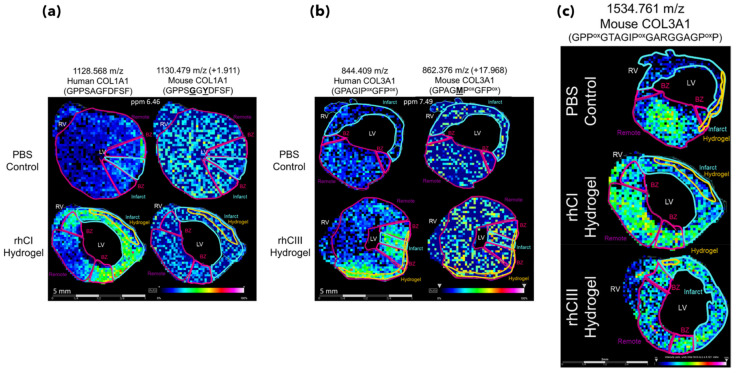
Mouse model study of the impact of collagen-based hydrogels on endogenous extracellular matrix (ECM) remodeling. Human-specific collagen sequence (left) and corresponding mouse sequence (right) in (**a**) collagen type 1 hydrogel in comparison with PBS control, and (**b**) collagen type 3 hydrogel in comparison with PBS control (**c**) Mouse-specific COL3A1 peptide differentially abundant in PBS control and in hydrogel treatment. Orange—hydrogel injection, blue—infarct region, red—border zone, purple—remote. BZ: border zone; LV: left ventricle; RV: right ventricle. Adapted with permission from American Chemical Society [78].

Moreover, the study allows us to identify the mouse ECM peptides participating in endogenous mouse ECM remodeling in response to hydrogel treatment. Examples of such peptides are presented in Figure 6. This study proved that MSI can analyze not only the biomaterial itself but also potential interactions with the treated tissue. Authors have shown that the approach using MSI is efficient and specific enough to serve as a detection method in the model of hydrogel-based therapies, giving a deep insight into the treatment process at a molecular level.

## 6. Mass Spectrometry Imaging of Biomaterials Used for In Vitro Cell Cultures

In vitro experiments provide us with a lot of knowledge about the basic biochemistry behind the cell’s interactions in the tissue and cells’ interactions with different materials and surfaces. Of course, we have to be aware that the picture drawn in that way could be simplified because we usually use only a single type of cell for this kind of experiment. It means that we are losing the complexity of the original tissue, but taking this fact into account allows us to draw the correct conclusions.

Properly designed peptides may be used to create a matrix that forces special kinds of cells to migrate in a given and desired direction. Moreover, specific sequences of amino acids grafted to synthetic or natural materials may promote cell survival, attachment, and proliferation and may influence the expression of specific receptors [81,82]. The MSI approach is very useful in characterizing such materials—since it does not demand any kind of labeling prior to the analysis. Moreover, in the case of MALDI analysis, intact chemical species on the analyzed surface may be identified.

**Figure 7:**
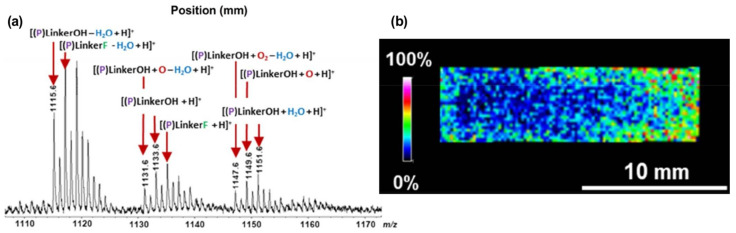
The gradient of CQAASIKVAV peptide profile confirmed by MALDI-MS analysis: (**a**) MS peaks corresponding to the tethered peptide mass and linker; (**b**) The MALDI MSI image corresponds to the *m*/*z* signal, which increases along the concentration gradient (reprinted with permission from Elsevier [83] with modifications).

Many cells use the chemical gradient to migrate in the desired direction. Among them, Schwann cells are in the spotlight because they are responsible for neuroregeneration after peripheral nerve injury. Motta et al. used a confined channel vapor deposition to create a concentration gradient of five different peptides on the microscope glass coverslips [83]. The selected sequences (RGD, PDSRG, YIGSR, IIKDI, IKVAV) were proven to be involved in peripheral nerve regeneration [81,84,85,86]. Their study assessed Schwann cell adhesion, proliferation, morphology, and migration parameters, relative to the peptide sequence, peptide concentration, and gradient slope. Multiple methods have been used to study generated surfaces (among them, contact angle measurement and X-ray Photoelectron Spectroscopy). MALDI MSI has been utilized to confirm the peptide gradient profile along the surface (see Figure 7).

In this case, it was not a classical MSI approach since the original surface was not prepared on the ITO glass. The procedure demanded the liberation of peptides from the glass by applying NH_4_F and covering the surface with a CHCA matrix using a thin-layer chromatography (TLC) sprayer. The obtained results provided evidence that the peptide gradient was preserved despite such a complicated preparation procedure.

In conclusion, this study proved that YIGSR showed a selective hypotactic effect on Schwann cells. More generally, such matrices with specific profiles of bioactive peptides may be used as therapeutic devices that facilitate functional recovery of the peripheral nerves [87]. Moreover, MALDI MSI analysis may be used here to confirm the correct surface preparation.

A slightly different approach for gradient preparation was proposed by Ender et al. [88]. They utilize a unique feature of amyloid-peptides functionalized with RGD motif to form a concentration gradient. Amyloid peptides, formerly associated with pathological conditions, are increasingly exploited as biomaterials. Those peptides are able to self-assemble into highly ordered fibrils with characteristic amyloid β-sheet structures. During this transformation, they gain special physical prosperities such as long-term stability, mechanical stiffness, and strong adhesion to various substrates [89,90,91]. In the mentioned study RGD motif was connected via a photocleavable linker (PLC) to the bioactive self-assembling peptide CKFKFQF [92]. In the physiological environment, the modified peptides (RGD-PCL-CKFKFQF) were self-assembled into the amyloid-like structure, and a gradient was created in a dose-dependent manner by surface exposure to UV light. Light irritation causes the RGD epitopes to cleave off and release, but the fibril morphology is maintained, which was confirmed by thioflavin T (ThT) assay, Fourier transform infrared spectroscopy (FT-IR), and TEM measurements.

**Figure 8:**
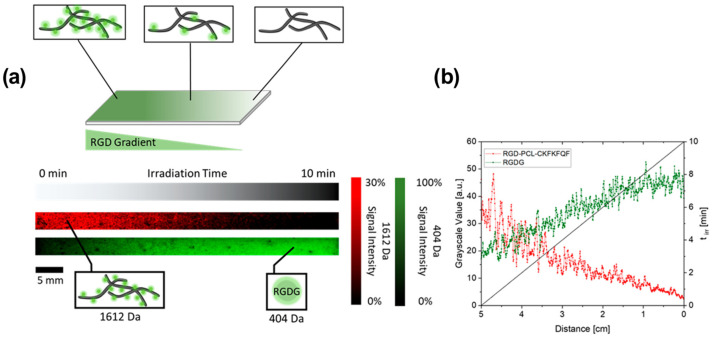
Gradual distribution of precursor (RGD-PCL-CKFKFQF—*m*/*z* 1613.7) and fragment ions (RGDG—*m*/*z* 404) (**a**) MALDI-MS analysis of the surface with the appropriate ion maps (**b**) Irradiation time dependent decrease in precursor signal and increase in RGDG-fragment (reprinted with modifications, with permission from ACS Publications [88].

MALDI—MSI was used for relative label-free quantitation of precursor (RGD-PCL-CKFKFQF—*m*/*z* 1613.7) and fragment ions (RGDG—*m*/*z* 404), based on the corresponding ions intensities in mass spectra. In this case, a separate sample was prepared where the peptide solution was spray-coated with an airbrush on the ITO glass surface. Then the RDG gradient was fabricated by UV-irradiation, and the CHCA matrix was used for the final analysis, which confirmed the gradual change in the signal 1613.7 *m*/*z* from a non-irridated surface relative to the irritated part (see Figure 8).

The analysis of the kinetics of photocleavage on the surface in the dry state was also possible due to the MALDI-MSI usage. The peptide fibril-coated ITO glass was irradiated with UV light for longer times (0, 1, 2, 3, 4, 5, 6, 8, and 10 min). Then the signal intensity of 1613.7 *m*/*z* from the irritated surface relative to the non-irritated surface was used as a quantitative measure for the remaining precursor, and the kinetics of the reaction could be established.

The MSI approach combined with in vitro studies, can also be used to analyze organic nanoparticles designed as potential drug carriers. ToF-SIMS is a method of choice in such studies, due to its high spatial resolution. Once again, as with all MSI techniques, it is label-free, so it does not need any kind of modification for the original nanoparticle. Moreover, SIMS allows for 3D analysis of the cells by using a sputter gun, thus the localization of the nanoparticles in the cell could be indicated. The problem with organic nanoparticles is that primary ions may cause their fragmentation, and obtained masses may be similar to cell components. Thus, analytical methods such as PCA have to be used to find the patterns characteristic of nanoparticles.

**Figure 9:**
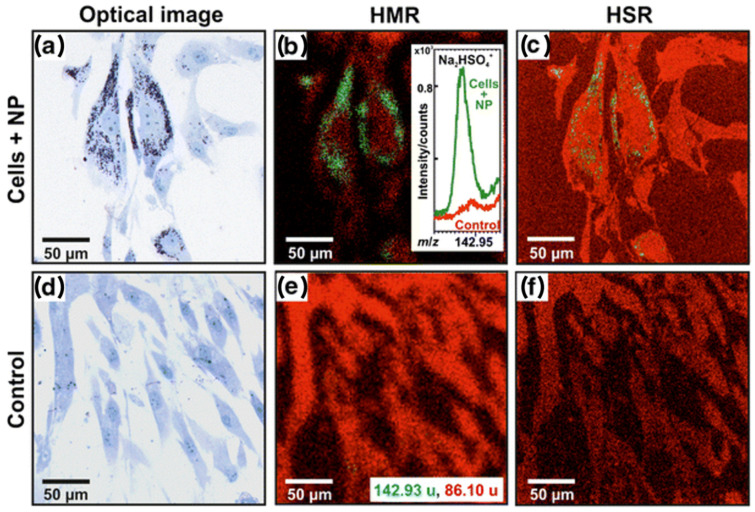
Distribution of PEI/CS–NP (**a**,**d**) optical image (**b**,**e**) high mass resolution (**c**,**f**) high spatial resolution. Reprinted with modifications, with permission from Springer [93].

In their study, Kokesch-Himmelreich et al. [93] focused on the analysis of polyelectrolyte complex nanoparticles made of polyethylenimine and cellulose sulfate (PEI/CS) in human bone marrow-derived stromal cells (hBMSC). The cells were cultured with those nanoparticles on silicon wafers to enable SIMS analysis. At the end of the procedure, the cells were washed with phosphate buffer saline (PBS) without Ca^2+^ and Mg^2+^, incubated with 4% paraformaldehyde for 10 min, and stored at 4 °C in PBS. Before the measurements, all samples were washed twice with pure water and measured using Bi^3+^ cluster in positive ion mode. To indicate specific masses for the PEI/CS nanoparticles, PCA analysis of the spectra of pure silicon wafer, PEI/CS nanoparticles alone, and chemically fixed control hBMSC was performed. The analysis indicated the *m*/*z* values that showed higher intensities in the nanoparticle mass spectra than in the cell-derived mass spectra. These values were used to show the distribution of nanoparticles in the cells cultured in their presence. Interestingly, only cellulose sulfate-related peaks (CS) and not nitrogen-related peaks (PEI) were indicated and could be used for the imaging experiments. These signals have relatively low intensities compared to the cell-derived signals, so a longer analyzing time is required to obtain reasonable images with high lateral resolution and good contrast. The study proved that PEI/CS–NP can enter hBMSC, and accumulate in the cytosol, and the signal from polymer-based nanoparticles may be distinguished in a cell environment from cell components. Nevertheless, the authors’ critical discussion indicates that classical toluidine blue-stained images of the cells, in this case, could be better than SIMS images (see Figure 9). This staining shows PEI/CS–NP are shown in dark blue due to a selective staining of acidic tissue components such as sulfates and carboxylates [94]. The disadvantages of the MSI approach here are related to lower sensitivity and the relatively small area that the SIMS analysis could cover compared with the staining method. This is an obstacle, especially when the cells do not incorporate nanoparticles evenly, and some do not have them at all. SIMS advantages can be seen in the possible 3D analysis of particle distribution that could be performed by surface sputtering with C_60_^+^.

## 7. Studying the Implant Formulation and Active Pharmaceutical Ingredient Release from Biomaterials Such as LAP and Microspheres with the Aid of Mass Spectrometry Imaging

Drug release profiles from polymeric implants depend both on polymer erosion and diffusion process. For this reason, the characterization of implant formulations is very important for understanding the active pharmaceutical ingredient (API) release mechanism and for all interactions with the polymeric matrix. The release profile of the drug might be limited by the formulation of the pharmaceutic or polymer coating and the dispersion process. Another aspect is the surface chemistry. The use of 3D chemical images allows the study of both surface and subsurface distribution of drug molecules and allows visualization of drug distribution as a function of elution time. Recent developments in MSI analytical techniques result in an additional dimension to the in-depth characterization of drug delivery systems. Mass spectrometry detection provides strict molecular information of various chemical compounds presented both on the surface of the biomaterial and in-depth [95].

Long acting parenterals (LAPs) implants are based on a drug delivery technology that enables the delivery of therapeutically effective doses of a drug over a wide range of time from weeks to years. Pierson et al. used DESI coupled with an ion mobility analyzer to study the spatial distribution of entecavir on the surface of cylindrical biodegradable poly(D,L-lactide) PLA implants without any special sample preparation. The DESI-MSI results indicate that there are differences in drug release between the two solutions. A more uniform release of entecavir from the implant was obtained using a low pH PBS solution, non-uniform release of entecavir was observed with methanol solution (MeOH: H_2_O (50:50, *v*/*v*)), and a higher relative concentration in the core of the implant. In addition, a decrease in entecavir concentration was observed from the surface of the implant to a diameter of approximately 1.5 mm from the center of the implant, while maintaining the maximum relative amount of entecavir at the center. This drug distribution shows that entecavir is mainly released from the outer surface of the implant. It is worth mentioning that the DESI-MSI images strictly depend on the intensity of the ion signal in the MS spectrum and two main aspects: the relative concentration of the drug in pixels, and the relative distance between the DESI capillary and the sample surface. Normalization as a function of ion intensity to the total ion chromatogram (TIC) of each pixel allowed for better relative comparisons of differences in drug distribution [96].

In recent years, a novel procedure based on the MALDI-MSI technique for the study of the drug release process in LAP implants was prepared. The release profile of Islatravir from the LAP implants at four in vitro release (IVR) points (0, 50, 75, 100%) was analyzed by MALDI-MSI. Islatravir belongs to the group of nucleoside reverse transcriptase translocation inhibitors and suppresses HIV replication and is recognized as a high-potency drug [97]. The milestone was the development of an innovative sample preparation protocol that could be applied to LAP implants for MALDI-MS imaging. This process is crucial to obtain the correct imaging of the surface of non-conductive samples necessary for further analysis of the kinetics of the drug release process. For such biomaterials, a 5 nm thin layer of platinum has to be deposited on the surface to avoid charge build-up during the imaging, before coating with DHB matrix. To study the spatial distribution of active pharmaceutical ingredient (API) in the rod, the distribution of ion intensity *m*/*z* 294.0999 (protonated) and *m*/*z* 316.0825 (sodiated) were observed at four drug release points. At 0% release, the intensities of the tested ions were evenly distributed across the LAP cross-section. At subsequent release points of 50% and 75%, a gradient of ion intensity distribution was observed, with higher ion intensity in the center and lower ion intensity at the edge of the rod. At 100% release, only a few or no API signals were detected at both *m*/*z* monitored. The results obtained at different drug release points show that in vitro drug release is a gradient-based process. The spatial distribution of API in the LAP implant obtained through the imaging technique, combined with quantitative analysis, may allow the proposal of a drug release mechanism and further development of LAP implants [98].

**Figure 10:**
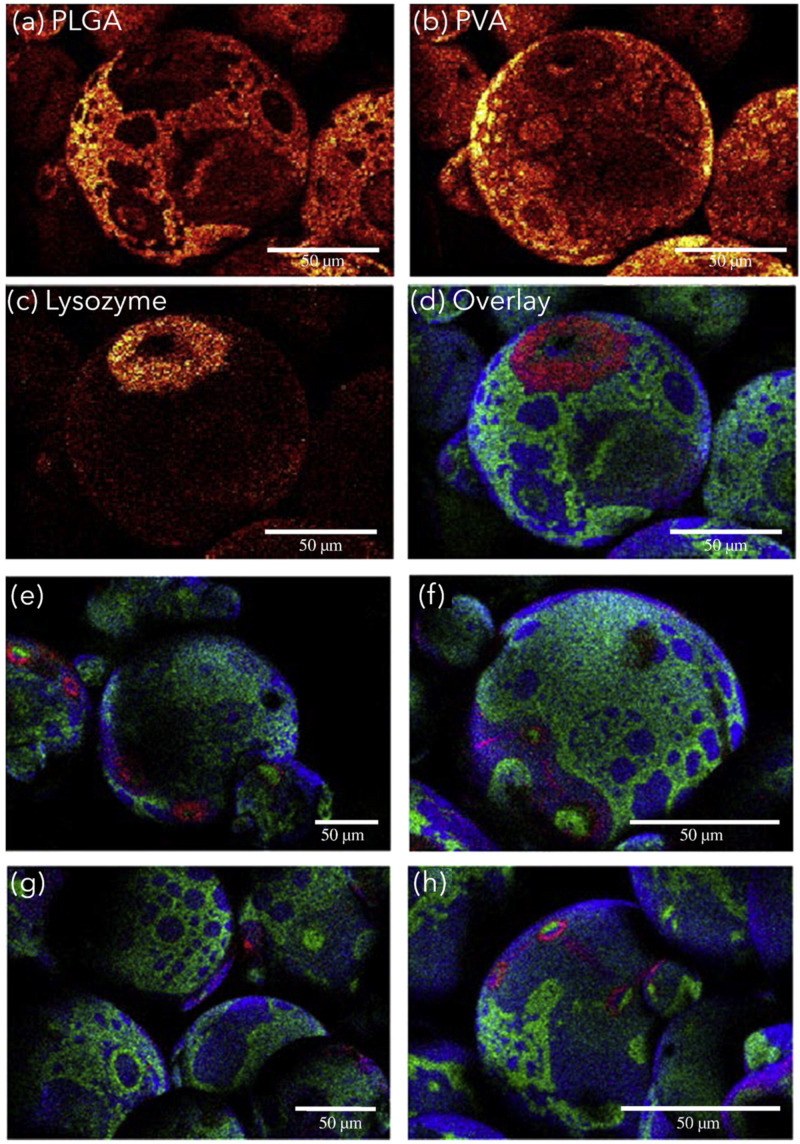
Images of ToF-SIMS analysis of the surface of the microsphere showing the secondary ion image which was generated from the identification anions from the ToF-SIMS analysis, for (**a**) PLGA (*m*/*z* 71/73, *m*/*z* 87/89), (**b**) PVA (*m*/*z* 59), (**c**) lysozyme (*m*/*z* 26, *m*/*z* 42), and (**d**) overlay which shows PVA (blue), PLGA (green), lysozyme (red). (**e**–**h**) overlay images of surface analysis by ToF-SIMS. Reprinted with permission from Elsevier [99].

Polymer microspheres enable the controlled release of various active substances The distribution of the drug in the microsphere has a very large impact on the release profile; therefore, the analysis of the microsphere volume is important for the pharmaceutical characterization of the system. The next stage of the research is to show the spatial location of the protein, surfactant, and polymer substrate using imaging of the surface of microparticles utilizing the AFM technique in combination with ToF-SIMS. Lysozyme released from PLGA microspheres was used as a protein-based model. The microspheres were prepared using a water-in-oil-in-water double emulsion method containing 1.5%, 3%, 5%, and 10% lysozyme. ToF-SIMS spectra were used for imaging the distribution of the PLGA, PVA, and lysozyme from the surface of the microspheres (Figure 10) based on reference ions (lysozyme (CNO^−^), PLGA (C_3_H_3_O_2_^−^, C_3_H_5_O_2_^−^, C_3_H_3_O_3_^−^, C_3_H_5_O_3_^−^), PVA (C_2_H_3_O_2_^−^)). The use of imaging techniques shows that the lysozyme on the surface of the microparticles was localized in different ways: most of the surface proteins appear to be densely clustered, which may be due to incomplete encapsulation of the protein-rich water droplets in the process of microparticle formation. There is also protein dispersed in the surfactant film itself, which may be due to an ionic interaction between the protein and the PVA. ToF-SIMS and Raman spectroscopy analysis has shown the presence of smaller pores (2–5 μm) containing protein dispersed in microparticles. In contrast, protein covers only the inner surface of larger pores (up to 20 μm), leaving most of the volume unoccupied. Through the use of both techniques, an overlap and volumetric analysis was performed, which can contribute to optimizing the therapeutic effect of a given treatment. The use of different fibers allowed this group to show the ability of ToF-SIMS to distinguish between chemicals and their complexity on the surface, through which it was shown that lysozyme is located in the pores formed by the evaporation of water from aqueous droplets dispersed in the organic phase of PLGA fibers. The presented studies have shown a complex and heterogeneous organization of protein distribution in microparticles, which may affect the mechanism and kinetics of protein release from microparticles [99].

## 8. Analysis of Designed Nanofibers by Mass Spectrometry Imaging

Long fibers with small diameters (50–500 nm) are called nanofibers. They can have many applications in the fields of biology, biomedical engineering, or cancer treatment. The main polymers used are polyurethane, polybenzimidazoles, polycarbonate, polyacrylonitrile, poly(vinyl alcohol), poly(lactic acid), poly(ethylene-co-vinyl acetate), poly(ethylene oxide), collagen, polyaniline, and poly(ethylene glycol); among them, silk, chitosan, and collagen, as well as poly(lactic-co-glycolic acid) (PLGA).

**Figure 11:**
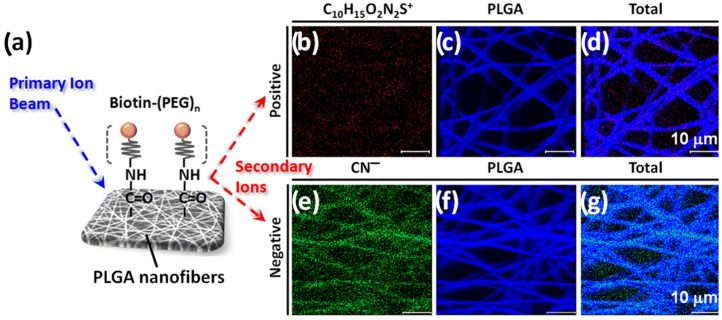
(**a**) Schematic of the biotin-(PEG)_n_ modified PLGA nanofiber surface which was prepared for the ToF-SIMS analysis. (**b**–**g**) ToF-SIMS chemical images of modified PLGA nanofibers in (**b**–**d**) positive ion mode for (**b**) C_10_H_15_O_2_N_2_S^+^, (**c**) PLGA nanofiber, (**d**) total ions and (**e**–**g**) negative ion mode for (**e**) CN^−^, (**f**) PLGA nanofiber, (**g**) total ions. Reprinted with permission from Springer Nature [100].

Among other applications, nanofibers can be used in the processes of capturing circulating tumor cells, which is possible due to the ability of nanofibers to fuse with cell matrices. Such a process is called electrospinning, and raw polymers and surface-modified polymers are used. Using the electrospinning process Yu et al. prepared PLGA nanofibrous arrays on glass slides [100]. They studied PLGA surface structures to improve the isolation of cancer cells from blood samples. During this process, special circulating tumor cells (CTCs) were used, which are important during cancer diagnosis, individual cancer therapy and cancer development. The authors modified special chips embedding the PLGA nanofiber arrays using sequential coating for the surface with many compounds, including biotin-(PEG)_7_-amine. They used the ToF-SIMS to study the surface modification of PEGylated biotin nanofibers [100]. Exemplary images are shown in Figure 11.

The possibility of studying the spatial distribution of different components using the ToF-SIMS was also presented by Scoutaris et al. [101]. In their study, they dealt with the polymer/API (active pharmaceutical ingredients) system, where they used polymer compounds (PVP and PLGA) as drug carriers and hydrochlorothiazide (HCT) and felodipine. They used inkjet printing of formulations of HCT/PLGA, HCT/PVP, and felodipine/PVP. Those formulations were printed as micro-dot arrays and analyzed by ToF-SIMS. There was heterogeneity in HCT/PLGA formulation, so multivariate curve resolution for the ToF-SIMS hyperspectral image dataset was applied. After that, chemical components: HCT, PLGA, substrate material, and many contaminants were identified. In order to mitigate the heterogeneity of the drug and other chemical components observed with the HCT/PLGA spots, PLGA was replaced with a PVP polymer. Images of secondary ions characteristic of both the hydrochlorothiazide and the polymer matrix obtained for the printed HCT/PVP spots showed a uniform surface appearance, confirming a homogeneous distribution. The use of PVP probably inhibits the mixing of the polymer observed in the case of HCT/PLGA spots, which may result from both different interactions of polymers with API and different droplet drying kinetics. In the PVP-based formulation where felodipine was used, a uniform distribution was observed [101,102]. These results show that using a PVP matrix is a more effective method for controlling drug release [101].

Goessl et al. used ToF-SIMS for detailed surface imaging of the pattern components working with plasma lithography, which is described as a versatile method to manufacture all-polymetric substrates with thin-film patterns [103]. The plasma lithography process could be divided into several steps. The first step is connected with the coating of the surface with a tetraglyme polymer. After that, photolithographic processing is started. At the beginning of this process, a photoresist spin-coating is performed, and then the surface is exposed to the UV, but with the aid of a photomask which is used to create a characteristic pattern of the substances on the modified surface. After that the third layer: fluorocarbon is placed by plasma deposition. The last step of the treatment is the lift-off process—where all the materials that were not in intimate contact with the surface are removed. For this process, PGMMA (propylene glycol monomethyl ether acetate), was used. Such prepared surface was analyzed by ToF-SIMS imaging to chemically analyze the components and their distribution on the surface [103].

## 9. Dialyzer Polymer Membranes and Modified Surfaces Analyzed by Mass Spectrometry Imaging

In modern biomaterials technology, very high requirements are placed on the designed materials. Therefore, there is a constant need to solve very complex problems as well as to refine existing solutions. In most cases, it is impossible to find one material that would combine all the requirements, such as biocompatibility, mechanical strength, biodegradability and promotion of cell adhesion, proliferation, and differentiation. There are two strategies that can at least partially solve these problems: the use of composite materials that combine the properties of various components, or the selective modification of the surface of the material using various modification techniques.

### 9.1. Dialyzer Polymer Membranes

Dialyzer is a model of an artificial kidney. It is used during dialysis treatment for patients with kidney diseases. The task of the dialyzer is to filtrate a patient’s blood via hundreds of hollow fiber membranes, from which it is made. Although this is a very important and useful treatment, it still needs improvement. One of those is the type of membrane that must be biocompatible and permeable. A high number of those features possess a polysulfone (PSf) membrane. Another type is an asymmetry cellulose triacetate (ATA) membrane, which has an enhanced asymmetric structure in terms of cross-sectional pore size. These types of membranes were used by Aoyagi et al. in their research on the evaluation of blood adsorption onto dialysis membranes by ToF-SIMS imaging [104]. They compared blood adsorption tendencies between PSf and ATA membranes, using ToF-SIMS and near-field infrared microscopy (NFIR). ToF-SIMS was used due to the possibility of obtaining detailed distributions of blood-related biomolecules thanks to the high sensitivity and ability to chemically map submicron-scale. MSI proved that there is blood adsorption on the inside surface of the PSf membrane in opposition to the ATA membrane, where there was almost no blood adsorption on the internal surface. Finally, the study shows the usefulness of imaging particular molecules (lipid- or protein-related secondary ions) in this kind of study [104].

Polysulfone membranes can be modified by adding hydrophilic compounds, such as polyvinylpyrrolidone (PVP). Biocompatibility is very important in the dialysis process, and it is influenced by polymer distribution. That is why compound identification and localization have a huge impact on membrane characterization. Biocompatible membranes cause the least inflammatory response in patients exposed. Polysulfone-based biomaterials are considered a gold standard for the production of biocompatible hemodialyzers. Firstly, MALDI imaging was used for investigating the chemical surface structure, by analyzing the distribution of the polymeric compounds on both sides of the membrane. The study showed that PVP was condensed in patches, and PS exhibited homogenous distribution at the abluminal (external) side. On the luminal side of the membrane, imaging showed that PVP was more homogenous than PS. Thus in this case, analysis of biocomponent membranes has shown differences in the composition of abluminal (external) and luminal (internal) membrane surfaces and polymer distribution [105]. Further research was made by the ToF-SIMS technique because this method is more surface sensitive. The group of Holzweber et al. has worked with those polymer membranes, using MSI with XPS to image investigated surfaces. Both techniques are useful and complementary tools for the characterization of polymer membrane surfaces [106]. The group analyzed flat membranes and hollow fiber membranes using ToF-SIMS to characterize surfaces of PS/PVP dialyzer membrane samples using positive and negative ion modes for imaging. ToF-SIMS analysis has shown “dark spaces”, where a lot of compounds related to preparation residues, were found. The MS images revealed differences between both sides of the dialyzer membrane. It has shown homogenous polymer distribution for analyzed surfaces, except for the abluminal side of the hollow fiber. Those analyses demonstrated that the MSI method could be a complementary method for in-depth profiling [106].

### 9.2. General Modified Surface Analysis

Modifying surfaces are more frequently used, especially those possessing carbohydrates (glycans) as a modification. Glycomics-based modifications have a lot of biomedical applications, like functional biomaterials or glycan-based biosensors. Carbohydrates are taking part in many biological processes. As it was mentioned, ToF-SIMS is a surface-sensitive technique, suitable for probing molecular composition at the surface of biomaterials. This feature was used by the Bolles et al. group with printed glycan microarrays surface, which was analyzed by ToF-SIMS imaging and Surface Plasmon Resonance imaging (SPRi) [107]. They used a microarray platform with located carbohydrate modifications. The use of those two techniques allowed us to examine glycoarray surface chemistries and bioactivity. ToF-SIMS was used to analyze individual spots on the arrays. During analysis, it was demonstrated that some molecules can be analyzed by ToF-SIMS, in the meantime being invisible in SPRi. The study provides that ToF-SIMS is relevant for the chemical characterization of modified surfaces [107].

The functionalization of nanoparticles enables the adjustment of interactions between them and the surrounding environment, which leads to obtaining nanostructured materials with a wide range of applications and, at the same time, gaining unique properties. Nanoparticles conjugated to biomolecules that can be used for biomimetics, targeted delivery or biosensing, are a key aspect in the design of new biomaterials. Shell cross-linked nanoparticles (SCK) are a group of polymeric nanostructured materials containing a hydrophobic core domain and a hydrophilic shell layer. Because they create stable nanoscale biocompatible scaffolds, they are a good starting point for further research and modifications [108]. In subsequent studies, biosynthetic hybrid multilayer structures composed of biotinylated nanoparticles with a cross-linked coating (SCK) on the streptavidin/biotin self-assembled monolayers (SAM) surface were built. At each stage of assembly, the chemistry and structure of the construct were monitored using a combination of ToF-SIMS (in positive and negative ion modes) and other imaging techniques. Prior to biotinylation, the ToF-SIMS images showed a uniform distribution of low ion intensities derived from SC_5_H_11_^+^, characteristic of self-assembled monolayers (SAM). After biotinylation, higher intensities of ions were observed, including C_4_H_9_O_2_N^+^, a characteristic fragment of biotinylated etylene glycol EG_6_COOH SAM. The data obtained have shown streptavidin absorption on the surface, mainly in biotin-rich areas. Moreover, surface-immobilized streptavidin was bioavailable and able to bind biotinylated SCKs. However, fluorescence microscopy images indicate that the biotinylated SAMs were not fully coated with streptavidin. In addition, biotinylated SCKs were shown to be significantly deformed as their cross-sectional diameter increased. The studies showed that functionalized SCKs can be used to investigate the factors controlling the adsorption of biomolecules on various functionalized surfaces [109].

The key aspect of good biointegration of an implant is the surface properties. Many biomaterials are porous, presenting analytical challenges compared to solid materials. The aim of the surface modification to receive a highly porous, fully fluorinated polymer substrate, so-called expanded poly(tetrafluoroethylene) (ePTFE), is to improve the biocompatibility and bioavailability of its surface which may lead to better biointegration. A number of specific modifications were used for this purpose. Graft copolymerization (induced by gamma irradiation) was performed using monoacryloxyethyl phosphate (MAEP) and methacryloxyethyl phosphate (MOEP) monomers in different solvent systems (methanol, water, methyl ethyl ketone and their mixtures). Detailed analysis of the grafted ePTFE membranes was obtained using various imaging techniques. Based on the received results, it was found that the copolymer penetration depth depends on the monomer, its concentration, the solvent used and the method of sample preparation. ToF-SIMS imaging was used to explore a 200 μm^2^ surface with a spatial resolution of ~120 nm. High-intensity ions were chosen for surface mapping. The F^−^ ion was used to represent the presence of the fluoropolymer substrate, while the PO_3_^−^ fragment was used to represent the grafted copolymer. By ToF-SIMS imaging, it was confirmed that the presence of graft copolymer was observed for all grafting modifications, especially in the fibrous regions of the membrane. This observation was most pronounced for samples with the MOEP in methyl ethyl ketone as the solvent. For samples grafted with MAEP monomers in methanol, the grafted copolymer was observed in all areas of the membrane. The combination of both techniques: ToF-SIMS imaging and microattenuated total reflectance Fourier transfer infrared (μ-ATR-FTIR) mapping provides the comprehensive information required to control the lateral distribution in the ePTFE substrate. Controlling the transverse distribution and penetration depth of the graft copolymer allows for the controlled production of bioactive ePTFE as a material that builds medical implants [110].

In the next study, the biocompatibility of membranes that are part of a bioartificial pancreas was investigated after its implantation in Goettingen mini-pigs. The surfaces of a polycarbonate membrane containing two polymers: polyvinylpyrrolidone (PVP), and hydroxypropyl methylcellulose (HPMC) were examined. After exposure in vivo, the membranes were analyzed by XPS and ToF-SIMS to investigate biomolecular adsorption, as well as surface degradation. Samples were tested at the beginning of the experiment and after 47 days from implantation in the intramuscular, subcutaneous zone, and abdominal wall. The images obtained during the analysis have shown that on the membrane treated with PVP, this molecule is still detected in the first layer of the surface. Such an image suggests that the attached biomolecular layer is very thin (thickness <1 nm) or only partially covers the surface. In other regions of the sample, ions from the PVP polymer were not observed, but fragments of biomolecules were seen, suggesting a thicker biomolecular layer. In the case of the HPMC-treated membrane, the HPMC signal decreased more on the external side than on the internal side of the membrane. On the outer surface, HPMC treatment is detected everywhere which means a very thin layer of attached biomolecules. An additional complication is the heterogeneity of the biomolecular layer. The presence of macromolecular compounds such as fatty acids, phosphates, and hydrocarbons were identified on both surfaces of the treated membranes. The results of ToF-SIMS imaging show the heterogeneity of biological molecules on the membrane surface in vivo. In conclusion, the biological molecules adhered to the surface of the encapsulating membrane after implantation, but the surface treatment chemistry remained unchanged [111].

## 10. Conclusions

The growing number of applications in recent years has proved the great potential of MSI for the analysis of biomaterials (see Supplementary Materials for a tabulation of the main aspects of articles discussed in this review). These methods offer unprecedented sensitivity, high specificity, and structural information capabilities, usually unattainable with other surface imaging techniques. Thus, along with other non-mass spectrometry-based analytical methods, scientists have a powerful tool to study biomaterials. As mentioned here, the great advantage of the MSI methods is their ability to indicate the chemical composition of the analyzed sample and to measure many different molecules in a single experiment simultaneously without any labeling. This enables us to learn and understand many biological issues.

MSI techniques are beneficial when the investigation of selected substances is important. It is clearly shown in the analysis of different biomaterials designed to elute active substances, such as drug-eluting stents, metal ion-eluting bone implants, or long-acting parenteral implants (LAP). Here, the analysis of elution of active substances from the material and its distribution in the surrounding tissue is important. Such research allows us to create kinetic models for the observed phenomena which may in the future allow us to obtain better materials and reduce the number of animal experiments [64]. Knowledge obtained in that way is crucial because in the future this kind of biomaterials may deliver drugs directly to where they should be active, replace inconvenient drug dosage, or reduce the amount of active substances that have to be administrated. It is worth mentioning that this kind of formulation may be preferred for the treatment of chronic diseases.

Another kind of study is devoted to analyzing the interaction of biomaterial with the surrounding tissues. Such investigations may indicate proteins and lipids that interact with the designed biomaterial. Once again obtained data may help to create biomaterials with better parameters adapted to their functions, since, in some circumstances, strong integration is welcome, and in others, not (for example, in the case of dialyzer membranes).

In conclusion, we cannot forget about the role of MSI in the quality control of designed materials produced by different techniques such as inject-printing or plasma lithography. Such materials may, in the future, revolutionize drug administration and act as a novel drug carrier that allows for personalized medicine formulation.

Nevertheless, biomaterials analysis could be challenging in a few ways, but the attempts to overcome these obstacles demonstrate the growing need for this type of analysis for biomaterials. First of all, classical MSI measurement is focused on the analysis of very thin samples placed on the conductive surface. Biomaterials could have much more complex shapes. Attempts to circumvent these obstacles demonstrate the growing need for this type of analysis for biomaterials. For example, Huang et al. [40] patented a special sample holding plate (USA, patent application No. # 20090294695) for 3D MALDI analysis of drug-eluting stents. Liang et al. developed a new method for measuring the samples that accumulate a charge, which is a problem for materials that are polymer-based, such as long-acting parenteral (LAP) implants [98]. They also designed a new MALDI holder to be able to measure the bulk section of an implant (1 cm long) since cutting it into thin slices on the cryotome at low temperatures was problematic. To avoid charge build-up on the surface of the LAP rod during the imaging run—a 5 nm thin layer of platinum was deposited on the surface of LAP implant samples. After that, a classical DHB matrix was used. The trend that is focused on adapting MALDI for biomaterials analysis also indicates the growing importance of such measurements. SIMS is a very versatile technique since, apart from the flat thin samples, more complex ones could be measured. To name a few: contact surface of the implant and bone [62], the section of microspheres [99], or nanofibers electrospun onto Al foil before the measurements [100]. However, the most significant limitation of SIMS measurements is high in-source fragmentation, and very often, finding characteristic peaks for analyzed substances demands PCA analysis. In contradiction, MALDI offers easier mass spectra interpretation and, once again, the attempts to adapt it for the biomaterials analysis indicate the usefulness of the knowledge obtained by this analysis.

Additionally, due to the constantly growing analytical capabilities of mass spectrometers, including spatial resolution, mass resolution, and scanning speed, it seems that their increasing use for the analysis of the surface of biomaterials is a foregone conclusion for the coming years.

## Data Availability

Not applicable.

## Supplementary Materials

The following supporting information can be downloaded at: https://www.mdpi.com/article/10.3390/ma16186343/s1, Table S1: A tabulation of the main aspects of articles discussed in this review [17,38,40,46,49,50,51,52,58,62,63,64,65,68,69,70,72,78,83,88,93,96,98,99,100,102,103,104,105,106,107,108,110,111].
