# Supplementary material for: Mass Spectrometry Imaging of Biomaterials
Source: Materials (Basel). 2023 Sep 21;16(18):6343. doi: 10.3390/ma16186343 (PMC10534324; doi:10.3390/ma16186343)
Supplement: Supplementary file 1 [file materials-16-06343-s001.zip › materials-2544362-supplementary.pdf]

Mass spectrometry imaging of biomaterials

Paulina Kret, Anna Bodzon-Kulakowska, Anna Drabik, Joanna Ner-Kluza, Piotr Suder and Marek Smoluch *

Department of Analytical Chemistry and Biochemistry, Faculty of Materials Science and Ceramics, AGH University of Krakow, A. Mickiewicza 30, 30-059 Krakow, Poland; pkret@agh.edu.pl (P.K.); anna.bodzon-kulakowska@agh.edu.pl (A.B.-K.); drabik@agh.edu.pl (A.D.); nerkluza@agh.edu.pl (J.N.-K.); piotr.suder@agh.edu.pl (P.S.)
* Correspondence: smoluch@agh.edu.pl

Supplementary materials

In the supplementary materials we would like to present a tabulation of the main aspects of articles discussed in the review such as the analyzed materials, MSI technique, analytes, and character of the results, which could be helpful for the interested readers.

Ref	Analysed item	Analyzed materials	technique	analytes
MSI of biomaterials in the vascular system: models and therapies				
[1]	drug-eluting stents (DES)	tissue sections	MALDI	everolimus – an eluted drug
[2]	drug-eluting stents (DES)	stents (cobalt-chromium scaffolding with different polymer coatings) and stented tissue area	MALDI	sirolimus an eluted drug, drug-related degradation products and polymer-related degradation products
[3]	drug-eluting stent coating composed of sirolimus in a PLGA matrix	alloy coupons (metal samples) coated with sirolimus in a PLGA	SIMS	sirolimus, PLGA (repeat units of lactic and glycolic acid)
[4]	drug-eluting stent coating composed of sirolimus in a PLGA matrix	MP35N metal alloy coupons and bare metal stents coated with sirolimus in a PLGA	SIMS	sirolimus ($C_5H_{10}N^+$ and CN^-), PLGA ($C_3H_4O^+$ and $C_3H_5O_2^-$)
[5]	drug-eluting stents (DES)	stents (cobalt-chromium scaffolding with different polymer coatings) and stented tissue area	SIMS	analysis of polymeric coatings and eluted drug
[6]	searching of the presence of collagen on the modified poloxamine matrix	the surface of collagen-modified poloxamine hydrogel	SIMS	different amino acid fragments, corresponding to the collagen, and PEO and PPO blocks
[7]	biodegradable vascular implants	expanded polytetrafluorethylene (ePTFE) and thermoplastic polyurethane (TPU) grafts	MALDI	protein and lipid deposition on polymers
[8]	vascular graft	polyethylene	DESI	lipids deposition inside

		terephthalate (Dacron) InterGard® vascular graft		and on the graft surface
MSI of bone implant biomaterials				
[9]	implant (titanium)-tissue (bone) interface: mineralization process	implat – tissue section (special kind of low-speed saw (Isomed™)	SIMS	hydroxyapatite fragment ions
[10]	brushite forming calcium phosphate cement with Cr^{3+}	implant region and the surrounding bone tissue	SIMS	calcium and chromium ions
[11]	strontium releasing bone cements	implant and healthy and osteoporotic rat bone tissue – Sr^{2+} diffusion	SIMS	calcium and strontium ions
[12]	synovial lipid adsorption on acetabular cup	Ultrahigh molecular weight polyethylene (PE-UHMW) materials (GUR-1050 and vitamin E-doped)	MALDI	lipids
[13]	bone and bone marrow	bone and fat tissue sections	OrbiSIMS	strontium ions, hydroxyapatite, fatty acids
[14]	titanium-niobium (Ti-^{40}Nb) implant with strontium surface functionalization	strontium-containing coatings composition	SIMS	different ions such as: SrO^+,$SrCl^+$,FeO^+,F^+, Ti^+, Nb^+,NbO_2^+,TiO_2^+, $^{37}Cl^-$, FeO^-,SrO^-,TiO^-,NbO^-,O_2^-, F_2^-
[15]	implantation of MgO (resorbable biomaterial) into bone marrow cavity	bone tissue sections	SIMS	Ca, Mg P and hydroxyapatite related species
[16]	analysis of impurities on ceramic oral implants	packaging material and implant surface	SIMS	talc, DBSA, and fatty acid ester
Evaluation of biomaterials influence on the adjacent tissue by MSI				
[17]	interaction between the biomaterial (hydrogel drug delivery carriers) and the tissue (renal capsule)	the sections of tissue with implanted hydrogel	SIMS	polymer-specific signals and biologically relevant peaks identified by PCA analysis
[18]	interaction between collagen hydrogel and non-contractile scar tissue in mice myocardium	the sections of tissue with implanted collagen hydrogel	MALDI	peptides
MSI of biomaterials used for in vitro cell cultures.				
[19]	observation of generated surfaces with peptide gradient for the cell culture	glass surface prepared for the cell culture with peptides liberated by applying NH_4F.	MALDI	MS peaks corresponding to the tethered peptide mass and linker
[20]	controlling the fabrication	fibril-coated and UV-	MALDI	peaks for precursor

	of gradient of integrin-binding peptide RDG on the surface for the cell culturing	irradiated surface of the ITO slide		peptide characteristic for non-irridatednand and microwave irritated surface (fragment ion)
[21]	confirmation of the presence of nanoparticles inside the cells *in vitro*	the cells were cultured with nanoparticles on silicon wafers	SIMS	cellulose sulfate-related peaks identified by PCA analysis
Studying the implant formulation and active pharmaceutical ingredient release from biomaterials such as LAP and microspheres with the aid of MSI				
[22]	elution of entecavir from polymeric implants containing 40% (w/w) entecavir and poly(D,L-lactide) (PLA)	the intact and radial implant sections	DESI	entecavir ions and contaminants
[23]	drug release process from non-conductive polymeric based LAP implants	LAP implants were smoothed using microtomes and measured with special holder	MALDI	Islatravir, sodiated and protonated
[24]	lysozyme released from PLGA microspheres	microspheres on double sided tape attached to aluminium blocks and their ultramicrotome sections	SIMS	reference ions for lysozyme and PVA
Analysis of designed nanofibers by MSI				
[25]	analysis of the surface of biotin-$(PEG)_n$ modified PLGA nanofibers	reticulated PLGA nanofiber arrays electrospun onto Al foil	SIMS	the fragment ions of PLGA and characteristic signals from the secondary ions of biotin–(PEG)7–amine
[26]	PLGA and PVP modified with hydrochlorothiazide (HCT) and felodipine micro-dots	HCT/polymer and felodipine/polymer printed spots	SIMS	ions characteristic of the hydrochlorothiazide, felodipine and the polymer matrix
[27]	polymers manufactured by plasma lithography	the analysis of homogeneous plasma polymers, and patterned surfaces	SIMS	ions characteristic of used substrates
Dialyzer polymer membranes and modified surfaces analyzed by MSI				
[28]	evaluation of blood adsorption onto dialysis	the hollow fiber membranes before and	SIMS	the known fragment ions of lipids proteins

	membranes	after blood circulation		and those related to polysulfone (PSf) or the main additive polyvinylpyrrolidone
[29]	characterization polymeric dialyzer membranes, consisting of polysulfone (PS) and polyvinylpyrrolidone (PVP)	membranes fixed with adhesive tape onto glass slides	MALDI	ions corresponds to polyvinylpyrrolidone and the polysulfone repeating unit
[30]	characterization polymeric dialyzer membranes, with polysulfone (PS) and polyvinylpyrrolidone (PVP)	surface of membranes	SIMS	the pyrrilidon side group $C_6H_{10}NO^+$, and $C_4H_6NO^-$, for PVP and repetition unit $C_{15}H_{14}O^+$ for PS
[31]	Surfaces covalently modified by carbohydrates	printed microarray chips	SIMS	ions characteristic for carbohydrates indicated by PCA
[32]	self-assembled monolayers (SAMs)	biotin–streptavidin–biotin SCK nanoparticle construct on Si wafers	SIMS	ions characteristic for self-assembled monolayers and biotinquali
[33]	analysis of the grafted expanded poly(tetrafluoroethylene) (ePTFE) membranes	expanded poly(tetrafluorethylene) (ePTFE) membrane	SIMS	the F^- ion - fluoropolymer substrate, the PO_3^- fragment represent the grafted copolymer
[34]	investigation of biomolecular adsorption on polyvinylpyrrolidone (PVP), and hydroxypropyl methylcellulose (HPMC) membrane	membranes after 11 and 42 days of implantation	SIMS	peaks characteristic of different biomolecules

1. Razzi, F.; Lovrak, M.; Gruzdyte, D.; Den Hartog, Y.; Duncker, D.J.; van Esch, J.H.; van Steijn, V.; van Beusekom, H.M.M. An Implantable Artificial Atherosclerotic Plaque as a Novel Approach for Drug Transport Studies on Drug-Eluting Stents. *Adv Healthc Mater* **2022**, *11*, e2101570, doi:10.1002/adhm.202101570.
2. Huang, J.-T.; Hannah-Qiuhua, L.; Szyszka, R.; Veselov, V.; Reed, G.; Wang, X.; Price, S.; Alquier, L.; Vas, G. Molecular Imaging of Drug-Eluting Coronary Stents: Method Development, Optimization and Selected Applications. *J Mass Spectrom* **2012**, *47*, 155–162, doi:10.1002/jms.2046.
3. Belu, A.M.; Graham, D.J.; Castner, D.G. Time-of-Flight Secondary Ion Mass Spectrometry: Techniques and Applications for the Characterization of Biomaterial Surfaces. *Biomaterials* **2003**, *24*, 3635–3653, doi:10.1016/s0142-9612(03)00159-5.
4. Mahoney, C.M.; Fahey, A.J.; Belu, A.M. Three-Dimensional Compositional Analysis of Drug Eluting Stent Coatings Using Cluster Secondary Ion Mass Spectrometry. *Anal Chem* **2008**, *80*, 624–632, doi:10.1021/ac701644j.

5. Belu, A.; Mahoney, C.; Wormuth, K. Chemical Imaging of Drug Eluting Coatings: Combining Surface Analysis and Confocal Raman Microscopy. *J Control Release* **2008**, *126*, 111–121, doi:10.1016/j.jconrel.2007.11.015.
6. Sosnik, A.; Sodhi, R.N.S.; Brodersen, P.M.; Sefton, M.V. Surface Study of Collagen/Poloxamine Hydrogels by a “deep Freezing” ToF-SIMS Approach. *Biomaterials* **2006**, *27*, 2340–2348, doi:10.1016/j.biomaterials.2005.11.028.
7. Fröhlich, S.M.; Eilenberg, M.; Svirkova, A.; Grasl, C.; Liska, R.; Bergmeister, H.; Marchetti-Deschmann, M. Mass Spectrometric Imaging of in Vivo Protein and Lipid Adsorption on Biodegradable Vascular Replacement Systems. *Analyst* **2015**, *140*, 6089–6099, doi:10.1039/c5an00921a.
8. Bodzon-Kulakowska, A.; Drabik, A.; Mystkowska, J.; Chlabicz, M.; Gacko, M.; Dabrowski, J.R.; Mielczarek, P.; Silberring, J.; Suder, P. Desorption Electrospray Ionization-Based Imaging of Interaction between Vascular Graft and Human Body. *J Biomed Mater Res B Appl Biomater* **2016**, *104*, 192–196, doi:10.1002/jbm.b.33385.
9. Eriksson, C.; Malmberg, P.; Nygren, H. Time-of-Flight Secondary Ion Mass Spectrometric Analysis of the Interface between Bone and Titanium Implants. *Rapid Commun Mass Spectrom* **2008**, *22*, 943–949, doi:10.1002/rcm.3445.
10. Rentsch, B.; Bernhardt, A.; Henß, A.; Ray, S.; Rentsch, C.; Schamel, M.; Gbureck, U.; Gelinsky, M.; Rammelt, S.; Lode, A. Trivalent Chromium Incorporated in a Crystalline Calcium Phosphate Matrix Accelerates Materials Degradation and Bone Formation in Vivo. *Acta Biomater* **2018**, *69*, 332–341, doi:10.1016/j.actbio.2018.01.010.
11. Rohnke, M.; Pfitzenreuter, S.; Mogwitz, B.; Henß, A.; Thomas, J.; Bieberstein, D.; Gemming, T.; Otto, S.K.; Ray, S.; Schumacher, M.; et al. Strontium Release from Sr2+-Loaded Bone Cements and Dispersion in Healthy and Osteoporotic Rat Bone. *J Control Release* **2017**, *262*, 159–169, doi:10.1016/j.jconrel.2017.07.036.
12. Fröhlich, S.M.; Archodoulaki, V.-M.; Allmaier, G.; Marchetti-Deschmann, M. MALDI-TOF Mass Spectrometry Imaging Reveals Molecular Level Changes in Ultrahigh Molecular Weight Polyethylene Joint Implants in Correlation with Lipid Adsorption. *Anal Chem* **2014**, *86*, 9723–9732, doi:10.1021/ac5025232.
13. Kern, C.; Jamous, R.; El Khassawna, T.; Rohnke, M. Characterisation of Sr2+ Mobility in Osteoporotic Rat Bone Marrow by Cryo-ToF-SIMS and Cryo-OrbiSIMS. *Analyst* **2022**, *147*, 4141–4157, doi:10.1039/d2an00913g.
14. Göttlicher, M.; Rohnke, M.; Moryson, Y.; Thomas, J.; Sann, J.; Lode, A.; Schumacher, M.; Schmidt, R.; Pilz, S.; Gebert, A.; et al. Functionalization of Ti-40Nb Implant Material with Strontium by Reactive Sputtering. *Biomater Res* **2017**, *21*, 18, doi:10.1186/s40824-017-0104-8.
15. Nygren, H.; Chaudhry, M.; Gustafsson, S.; Kjeller, G.; Malmberg, P.; Johansson, K.-E. Increase of Compact Bone Thickness in Rat Tibia after Implanting MgO into the Bone Marrow Cavity. *J Funct Biomater* **2014**, *5*, 158–166, doi:10.3390/jfb5030158.
16. Duddeck, D.U.; Albrektsson, T.; Wennerberg, A.; Larsson, C.; Mouhyi, J.; Beuer, F. Quality Assessment of Five Randomly Chosen Ceramic Oral Implant Systems: Cleanliness, Surface Topography, and Clinical Documentation. *Int J Oral Maxillofac Implants* **2021**, *36*, 863–874, doi:10.11607/jomi.8837.
17. Klerk, L.A.; Dankers, P.Y.W.; Popa, E.R.; Bosman, A.W.; Sanders, M.E.; Reedquist, K.A.; Heeren, R.M.A. TOF-Secondary Ion Mass Spectrometry Imaging of Polymeric Scaffolds with Surrounding Tissue after in Vivo Implantation. *Anal. Chem.* **2010**, *82*, 4337–4343, doi:10.1021/ac100837n.

18. Clift, C.L.; McLaughlin, S.; Muñoz, M.; Suuronen, E.J.; Rotstein, B.H.; Mehta, A.S.; Drake, R.R.; Alarcon, E.I.; Angel, P.M. Evaluation of Therapeutic Collagen-Based Biomaterials in the Infarcted Mouse Heart by Extracellular Matrix Targeted MALDI Imaging Mass Spectrometry. *J. Am. Soc. Mass Spectrom.* **2021**, *32*, 2746–2754, doi:10.1021/jasms.1c00189.
19. Motta, C.M.M.; Endres, K.J.; Wesdemiotis, C.; Willits, R.K.; Becker, M.L. Enhancing Schwann Cell Migration Using Concentration Gradients of Laminin-Derived Peptides. *Biomaterials* **2019**, *218*, 119335, doi:10.1016/j.biomaterials.2019.119335.
20. Ender, A.M.; Kaygisiz, K.; Räder, H.-J.; Mayer, F.J.; Synatschke, C.V.; Weil, T. Cell-Instructive Surface Gradients of Photoresponsive Amyloid-like Fibrils. *ACS Biomater. Sci. Eng.* **2021**, *7*, 4798–4808, doi:10.1021/acsbiomaterials.1c00889.
21. Kokesch-Himmelreich, J.; Woltmann, B.; Torger, B.; Rohnke, M.; Arnhold, S.; Hempel, U.; Müller, M.; Janek, J. Detection of Organic Nanoparticles in Human Bone Marrow-Derived Stromal Cells Using ToF–SIMS and PCA. *Anal Bioanal Chem* **2015**, *407*, 4555–4565, doi:10.1007/s00216-015-8647-9.
22. Pierson, E.E.; Midey, A.J.; Forrest, W.P.; Shah, V.; Olivos, H.J.; Shrestha, B.; Teller, R.; Forster, S.; Bensussan, A.; Helmy, R. Direct Drug Analysis in Polymeric Implants Using Desorption Electrospray Ionization - Mass Spectrometry Imaging (DESI-MSI). *Pharm Res* **2020**, *37*, 107, doi:10.1007/s11095-020-02823-x.
23. Liang, Z.; Giles, M.B.; Stenslik, M.J.; Marsales, M.; Ormes, J.D.; Seto, R.; Zhong, W. Direct Visualization of the Drug Release Process of Non-Conductive Polymeric Implants via Molecular Imaging. *Analytica Chimica Acta* **2022**, *1230*, 340395, doi:10.1016/j.aca.2022.340395.
24. Rafati, A.; Boussahel, A.; Shakesheff, K.M.; Shard, A.G.; Roberts, C.J.; Chen, X.; Scurr, D.J.; Rigby-Singleton, S.; Whiteside, P.; Alexander, M.R.; et al. Chemical and Spatial Analysis of Protein Loaded PLGA Microspheres for Drug Delivery Applications. *J Control Release* **2012**, *162*, 321–329, doi:10.1016/j.jconrel.2012.05.008.
25. Yu, C.-C.; Chen, Y.-W.; Yeh, P.-Y.; Hsiao, Y.-S.; Lin, W.-T.; Kuo, C.-W.; Chueh, D.-Y.; You, Y.-W.; Shyue, J.-J.; Chang, Y.-C.; et al. Random and Aligned Electrospun PLGA Nanofibers Embedded in Microfluidic Chips for Cancer Cell Isolation and Integration with Air Foam Technology for Cell Release. *J Nanobiotechnology* **2019**, *17*, 31, doi:10.1186/s12951-019-0466-2.
26. Scoutaris, N.; Alexander, M.R.; Gellert, P.R.; Roberts, C.J. Inkjet Printing as a Novel Medicine Formulation Technique. *J Control Release* **2011**, *156*, 179–185, doi:10.1016/j.jconrel.2011.07.033.
27. Goessl, A.; Garrison, M.D.; Lhoest, J.B.; Hoffman, A.S. Plasma Lithography--Thin-Film Patterning of Polymeric Biomaterials by RF Plasma Polymerization I: Surface Preparation and Analysis. *J Biomater Sci Polym Ed* **2001**, *12*, 721–738, doi:10.1163/156856201750411620.
28. Aoyagi, S.; Abe, K.; Yamagishi, T.; Iwai, H.; Yamaguchi, S.; Sunohara, T. Evaluation of Blood Adsorption onto Dialysis Membranes by Time-of-Flight Secondary Ion Mass Spectrometry and near-Field Infrared Microscopy. *Anal Bioanal Chem* **2017**, *409*, 6387–6396, doi:10.1007/s00216-017-0578-1.
29. Krueger, K.; Terne, C.; Werner, C.; Freudenberg, U.; Jankowski, V.; Zidek, W.; Jankowski, J. Characterization of Polymer Membranes by MALDI Mass-Spectrometric Imaging Techniques. *Anal Chem* **2013**, *85*, 4998–5004, doi:10.1021/ac4002063.

30. Holzweber, M.; Lippitz, A.; Krueger, K.; Jankowski, J.; Unger, W.E.S. Surface Characterization of Dialyzer Polymer Membranes by Imaging ToF-SIMS and Quantitative XPS Line Scans. *Biointerphases* **2015**, *10*, 019011, doi:10.1116/1.4907937.
31. Bolles, K.M.; Cheng, F.; Burk-Rafel, J.; Dubey, M.; Ratner, D.M. Imaging Analysis of Carbohydrate-Modified Surfaces Using ToF-SIMS and SPRi. *Materials (Basel)* **2010**, *3*, 3948–3964, doi:10.3390/ma3073948.
32. Qi, K.; Ma, Q.; Remsen, E.E.; Clark, Christopher G.; Wooley, K.L. Determination of the Bioavailability of Biotin Conjugated onto Shell Cross-Linked (SCK) Nanoparticles. *J. Am. Chem. Soc.* **2004**, *126*, 6599–6607, doi:10.1021/ja039647k.
33. Chandler-Temple, A.F.; Wentrup-Byrne, E.; Griesser, H.J.; Jasieniak, M.; Whittaker, A.K.; Grøndahl, L. Comprehensive Characterization of Grafted Expanded Poly(Tetrafluoroethylene) for Medical Applications. *Langmuir* **2010**, *26*, 15409–15417, doi:10.1021/la1010677.
34. Henry, M.; Ulrichs, K.; Moskalenko, V.; Bonneau, M.; Kang, C.; Belcourt, A.; Bertrand, P. Surface Analysis of an Encapsulation Membrane after Its Implantation in Mini-Pigs. *Biomed Mater* **2007**, *2*, S78-89, doi:10.1088/1748-6041/2/1/S12.
